# Health indicators on adolescents reveal disparity and inequality on regional and national levels

**DOI:** 10.1186/s12889-021-10989-1

**Published:** 2021-05-28

**Authors:** Mengqiao Wang

**Affiliations:** Department of Epidemiology and Biostatistics, West China School of Public Health and West China Fourth Hospital, Renmin South Road 16, Chengdu, Sichuan Province 610041 People’s Republic of China

## Abstract

**Background:**

Health status in adolescents is difficult to evaluate and compare horizontally, vertically and longitudinally among different regions and nations of the world.

**Methods:**

With repeated surveys conducted with relatively uniformed standards, the UNICEF Data warehouse compiles and publishes a wide spectrum of health indicators, of which data analysis and visualization would reveal the underlying statuses and trends on global, regional and national levels.

**Results:**

Apparent geographic disparity is present in that sub-Saharan African countries lag far behind their counterparts in other regions with regard to most health indicators on adolescents. Education attendance rates sequentially drop from primary to secondary school levels, and display correlation with youth literacy. Harmful practices of early marriage, early childbearing and female genital mutilation have decreased but the presences of peer violence and sexual violence are worthy of attentions. Although incidence and mortality rates of HIV/AIDS have dropped (most notably in sub-Saharan Africa), adolescents’ HIV/AIDS awareness remains suboptimal in selected countries. Cumulative COVID-19 cases and deaths in the adolescents are comparable to the children but relatively lower than the adult and senior groups.

**Conclusions:**

Findings on the health indicators of adolescents until 2019 reveal the most recent status quo for reference right before the hit of ongoing COVID-19 pandemic. Progresses made on the various health indicators as well as the associated disparity and inequality underlie the remaining gaps to fill for the achievement of the Sustainable Development Goals by 2030.

**Supplementary Information:**

The online version contains supplementary material available at 10.1186/s12889-021-10989-1.

## Introduction

Monitoring the health status of adolescents is important because health matters as a direct determinant of subsequent productivity and later well-being [1, 2]. The assessment and evaluation of both cross-sectional levels and longitudinal trends in adolescent health require evidence-based analysis of timely and readily accessible health indicators [3, 4]. The UNICEF Data warehouse compiles and releases standardized measures yielding information on the health of young populations on different dimensions of global, regional and national levels [5, 6]. Looking back at the developments in the past years towards universal target goals and currently facing an unprecedented COVID-19 pandemic, the time is ripe for a comprehensive review of the health indicators on adolescents.

In 2000, the United Nations adopted the Millennium Development Goals (MDGs) [7, 8], a blueprint agreed to by all of the world’s countries to address key determinants of human health and welfare. MDGs range from halving extreme poverty rates to halting the spread of HIV/AIDS and providing universal primary education, and for 15 years, the MDGs drove progress in these important areas. However, by the target year of 2015, not all goals were fully met in reality, including the targets on adolescent health. Nevertheless, for a post-2015 development agenda, all member states of the United Nations adopted in 2015 the Sustainable Development Goals (SDGs) that replace MDGs as a universal call to achieve 17 interconnected goals addressing the social, economic and environmental dimensions of sustainable development by 2030 [9–11]. Attached to the SDGs (https://sdgs.un.org/) are 169 concrete targets measured by 232 specific indicators (https://unstats.un.org/sdgs/metadata), among which at least 35 indicators are directly related to adolescents, e.g. ending the epidemics of AIDS, tuberculosis, malaria and neglected tropical diseases (Indicator 3.3), providing universal access to sexual and reproductive health-care services (Indicator 3.7), and ensuring free, equitable and quality primary and secondary education leading to relevant and effective learning outcomes (Indicator 4.1). Several obstacles are present against fulfilling SDGs in the young generation: 1). not all countries have sufficient data to assess the progress on track to achieve the SDG targets; 2). available data show an alarming number of countries needing to speed up progress to reach global targets; 3). geographical, gender and socioeconomic inequalities leave particular groups (poorest households, rural areas, females etc.) at greater risk of being left behind. A systematic retrospective and perspective analysis on the hierarchical dimensions of regional and national levels would thus be needed for the understanding of the most recent status (until 2019) and for the institution of the next-decade strategies (nearly 10 more years until 2030).

The proposal and implementation of SDGs as the blueprint to achieve a better and more sustainable future for all human beings have taken into account of major factors and uncertainties to designate a path of developments from 2015 to 2030. However, the United Nations have never expected the hit of a global pandemic. COVID-19 is a novel type of coronavirus appearing since late 2019, and the ongoing pandemic has resulted in over 135 million cases and 3 million deaths worldwide by April 2021 [12, 13], ranking COVID-19-associated pneumonia as one of the most devastating infectious diseases in the history of public health. While the full extent of COVID-19’s impact on economies, societies and health is still unknown but unfolding every day, the pandemic is destined to cause significant interruptions to the SDGs [14] and particularly the health of adolescents worldwide [15, 16]. While cumulative cases and deaths are the direct consequences of the pandemic, indirect influences from the many unprecedented COVID-19-related disruptions on health and human beings may not be readily measurable for some time until the pandemic recedes, and may even reverberate for an extended period following the end of pandemic [17, 18]. The severity of these indirect effects and the ability to mitigate them would significantly depend on socioeconomic status and pre-pandemic household conditions, so the indirect impacts of COVID-19 on the health of adolescents are likely to vary considerably on the global, regional and national levels. Therefore, this study aims to understand the status quo of health indicators on adolescents until pre-pandemic 2019, establishing the baseline and background to evaluate the negative impacts of COVID-19 on SDGs when peri-pandemic data of health indicators become available in future.

## Methods

### Data sources

Data of health indicators used in this study are publicly accessible from the UNICEF Data warehouse (https://data.unicef.org/dv_index/), last accessed in January 2021. Where possible, data are disaggregated by stratifying factors including geography (global, regional, national), residence (urban, rural), sex (male, female), education (school stages), age (age groups), wealth (5 wealth quantiles) etc. With regard to regional classifications by the UNICEF (https://data.unicef.org/regionalclassifications/), the world is segregated into a total of 9 regions with names and acronyms as: 1. East Asia and Pacific (EAP), 2. South Asia (SA), 3. Eastern Europe and Central Asia (EECA), 4. Western Europe (WE), 5. North America (NA), 6. Latin America and Caribbean (LAC), 7. Middle East and North Africa (MENA), 8. West and Central Africa (WCA), and 9. Eastern and Southern Africa (ESA). WCA and ESA together comprise the sub-Saharan Africa. Countries are annotated by the uniquely assigned three-character ISO 3166-1 alpha-3 codes (ISO3). For reference, the relative geographic map of all nations/territories is demonstrated in Fig. S1A (displayed not in exact scale to the physical world map). The countries/territories in the datasets refer to the United-Nations-designated sovereignties or entities, but should not be interpreted with regard to any legal status of territorial disputes. Divisions of development stages and age groups are not absolute, but a general definition is used as an approximate reference: children (aged 1–9), adolescents (aged 10–19) and youths (aged 20–24).

Data availability of raw UNICEF health indicators included in this study for individual countries/territories by respective regions is displayed in Fig. S1B. Overall, the developing countries are better represented than the developed countries regarding data coverage. Due to the incomplete coverage on all nations/territories, readers should interpret the discoveries and conclusions of this study as relevant to those countries represented. For those countries of which the corresponding UNICEF health indicators are unavailable, inquiry into national government statistics or academic literatures may help. However, this study strictly analyzes raw data available from the UNICEF Data warehouse. Similarly due to the issue of data coverage, not all regions are reported for certain indicators since data are not available for regions where population coverage is below 50%.

Data on the age-structured cumulative COVID-19 cases and deaths are retrieved from COVerAGE-DB [19] (a database of COVID-19 cases and deaths by age, https://osf.io/mpwjq/).

### Data analysis

For bivariate analysis of quantitative indicators, all countries are treated equally, visualized as individual points in the scatter plot, and reported with Pearson correlation coefficient (*r*), linear regression coefficient (*b*), and the null-hypothesis test *P* value (*P*). Trend lines are fitted by ordinary least squares linear regression of the corresponding points, using the *stats::lm()* function of R software. Data analysis and visualization are performed in version 4.0.2 of the R software (R Core Team, 2020) with assistance of several extended packages (*tidyverse*, *scales*, *geofacet*, *patchwork* etc.). Significance level of 0.05 is used in the null-hypothesis tests.

## Results

**Fig. 1:**
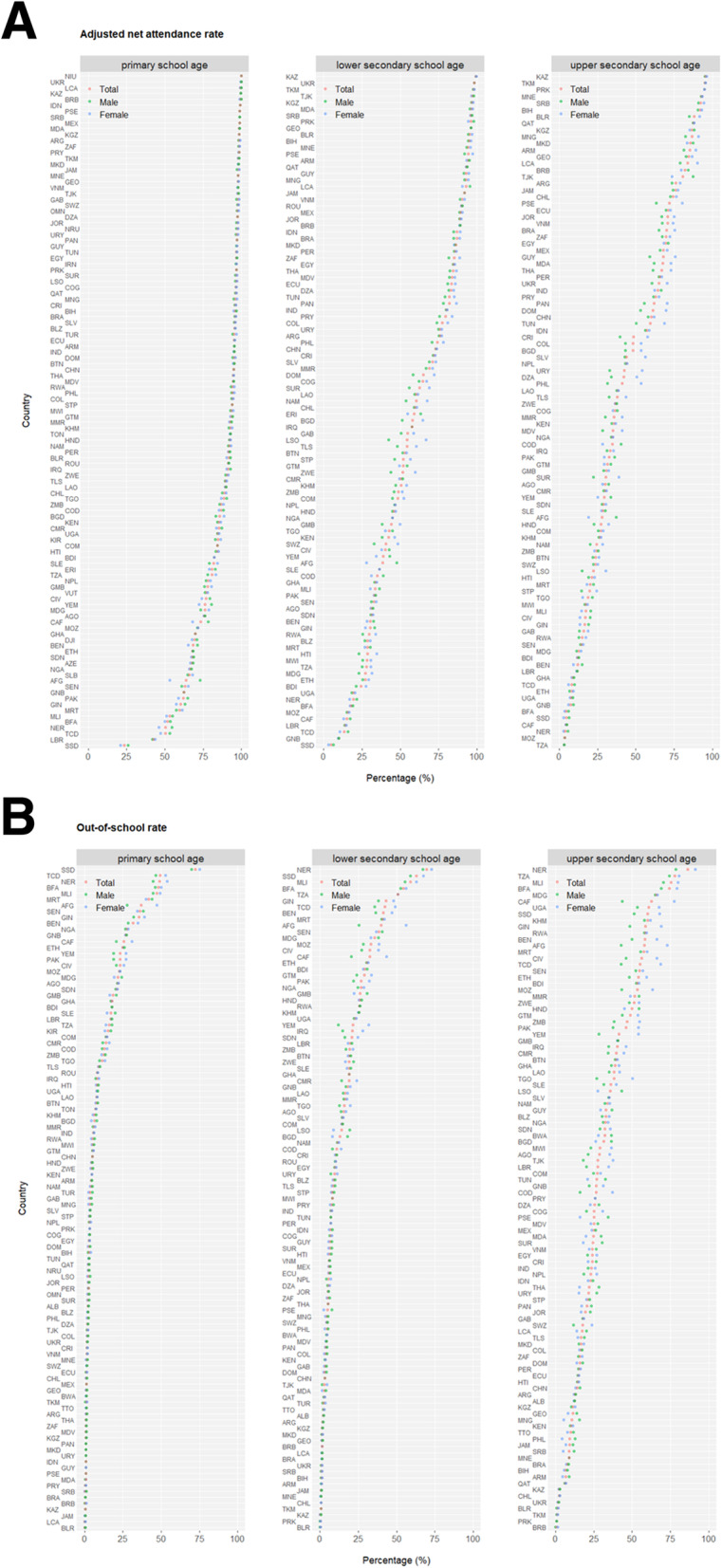

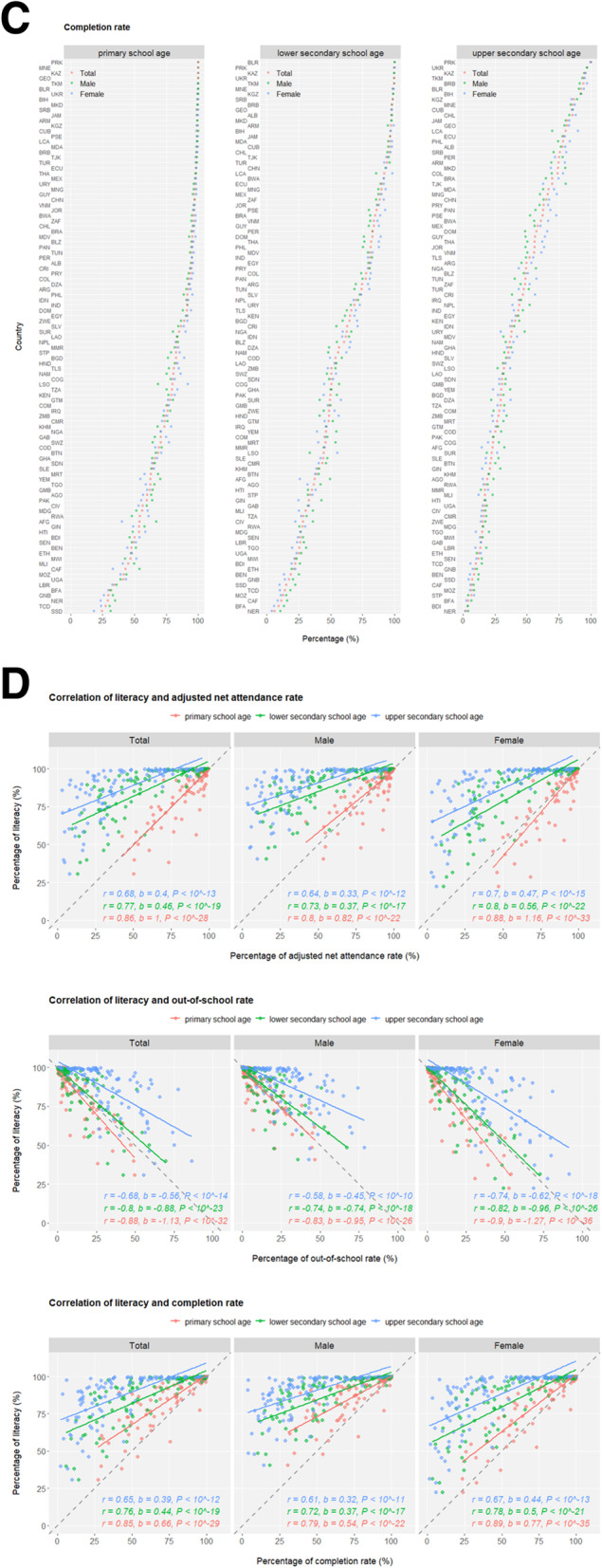
*(Continued)*

Demographics in the world display a wide spectrum of geographic disparities for the proportion of population under age 18 (highest of Niger at 56.7% and lowest of Singapore at 15.1%) (Fig. S2), and there exists strong correlation between population compositions under age 5 and under age 18, segregating nations into aging vs. blooming subgroups predominantly influenced by the associated total fertility rate (Fig. S3). Throughout childhood and adolescence, school education becomes a central component of life, building strong foundations for young people’s personal, social and overall well-beings into the adulthood. At least 51 (44.3%) out of 115 monitored nations have already achieved universal primary education (net attendance rate over 95%) but meanwhile at least one out of every four children are not yet enrolled in primary education in 22 (19.1%) countries (Fig. 1a). Along the sequential transition from primary to lower and upper secondary education, a general trend of “lower attendance rate, higher out-of-school rate, lower completion rate, and larger gender disparity” stands but significant disparities exist among nations with the worst performing ones predominantly mapped to sub-Saharan Africa (Fig. 1b and c). Interestingly, while no apparent gender disparity is observed in the attendance rates of primary school, higher rates of girls are enrolled in secondary level education compared to boys. Nationally, education parameters display high correlation between the two sexes (Fig. S4), but for the limited number of countries of which youth literacy rate is suboptimal (below 75%), literacy in females is consistently lower than in males (Fig. S5), underlying the potential gender inequality. Importantly, youth literacy correlates well with primary and secondary education status: primary education has the most profound effect on literacy (larger values of linear coefficient *b* and correlation coefficient *r*), and the relationship between primary/secondary education and literacy is more significant in females than males (larger *b* and *r*) (Fig. 1d), demonstrating the necessity for future efforts and interventions to promote fundamental education and remove gender disparity in adolescents.

**Fig. 2:**
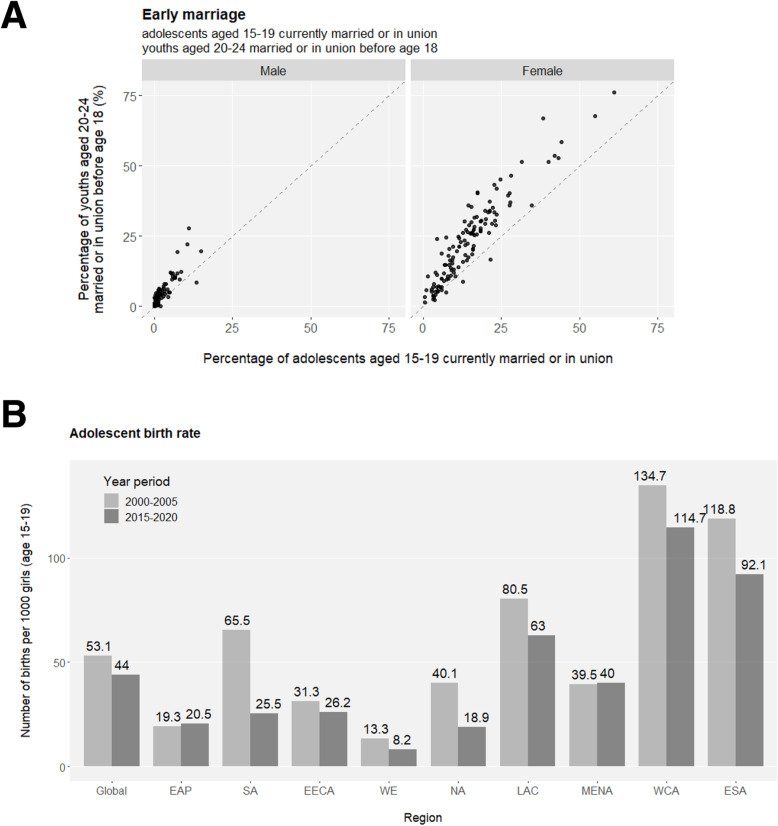

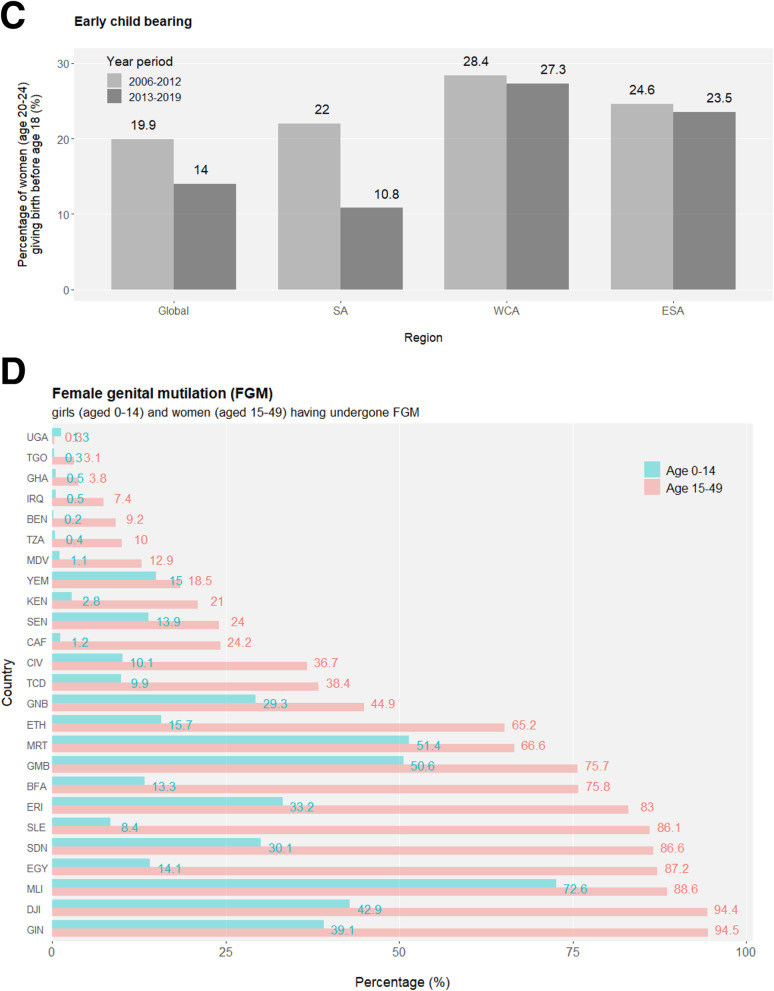
*(Continued)*

The transition from adolescence to adulthood spans hardships for health development. An unneglectable percentage of young people are bullied in the format of peer violence (Fig. S6), and female adolescents are especially vulnerable to sexual violence (Fig. S7). Considering their unwillingness or fear to report various forms of violence, the magnitude and nature of external development pressures on adolescents may be underestimated in reality, let alone the many types of internal difficulties they struggle with such as depression, suicidal thoughts etc. Early marriage (e.g. before age 18) and the unavoidably affiliated early childbearing are fundamental violations of adolescents’ human rights, particularly compromising girls’ personal developments, education opportunities and vocational advancements. Across the globe, levels of early marriage are significantly higher in females than in males, but there is a promising tendency of declines in both sexes demonstrated by the lower marriage-by-age-18 levels in the younger generation (age 15–19) compared to their older peers (age 20–24) (Fig. 2a and S8). Nevertheless, more than a quarter of female adolescents are married or coinhabited in a few sub-Saharan African countries, and globally, at least 12 million girls marry before their 18th birthday annually, setting great obstacles for the SDG Target 5.3 to “**eliminate all harmful practices, such as child, early and forced marriage**” in all countries by 2030. Adolescent birth rates observe overall decreases across regions but remain unacceptably high (Fig. 2b and S9A). Similarly, the highest proportion of early childbearing in female youths is mapped to sub-Saharan Africa (Fig. 2c). Inequality is eloquently present in that rural and poorest females have the highest likelihood of early childbearing (Fig. S9B). As another harmful practice aimed for complete elimination by 2030 (SDG Target 5.3), female genital mutilation (FGM) is a serious violation of female human rights, and it is estimated that at least 200 million girls and women have suffered from FGM. Highly concentrated in a swath of countries, the pace of overall declines in FGM has been uneven geographically (Fig. 2d).

**Fig. 3:**
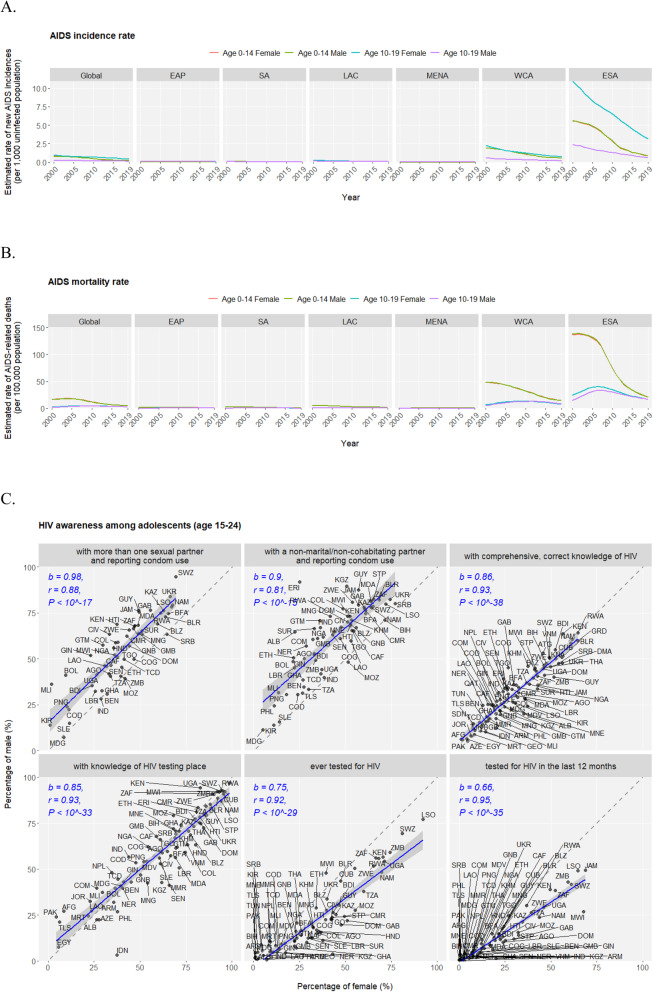
HIV prevention and treatment in adolescents. Annual AIDS incidence rate (new incidences per 1000 uninfected population) (**a**) and mortality rate (deaths per 100,000 population) (**b**) conditioned on age groups (“Age 0–14” and “Age 10–19”) and sex (male and female) in the globe and across regions during the past two decades (2000–2019). The time series are displayed as lines of annual rates in facted figures of regions; the four “age group - sex” levels are differntially colored. Note: data of HIV/AIDS epidemiology are available on only two age groups of 0–14 and 10–19 in the UNICEF Data warehouse, leading to analysis on these two partially overlapping rather than ideally exclusive age gorups. **c**. Scatter plots of six HIV awareness indicators in male vs. female among countries. Trend lines fitted by linear regression are displayed as blue solid lines with grey 95% confidence inetrvals. The corresponding Pearson correlation coefficient (*r*), linear coefficient (*b*) and null-hypothesis test P value (*P*) are reported in blue texts. Points corresponding to individual countries are annotated by the ISO3 codes. *Data source: UNAIDS 2020 estimates*

Human immunodeficiency virus (HIV) is responsible for causing acquired immune deficiency syndrome (AIDS) against which the world (especially sub-Saharan Africa) has battled for three decades achieving only progress but no victory. Adolescents continue to be seriously affected by the HIV/AIDS epidemics as an estimate of 1.7 million adolescents are among the approximately 38 million HIV-infected individuals by 2019. In the past two decades, AIDS incidence rates in adolescents declined across all regions, but ESA ranks the worst-hit region followed by WCA (Fig. 3a). Nearly all HIV infections in children under age 14 are caused by vertical transmission (from mother to child during pregnancy, delivery, or breastfeeding), and indeed no incidence difference could be observed between two sexes in the 0–14 age group. In sharp contrast, compared to the male, the female adolescents (10–19 age group) have much higher AIDS incidence rate (new infections per 1000 uninfected population) in ESA (female of 3.18 vs. male of 0.64 in 2019) and WCA (female of 0.74 vs. male of 0.20 in 2019), revealing the disproportionally higher risk among female teenagers in sub-Saharan Africa with regard to HIV exposures. Meanwhile, an estimate of 46,000 adolescents are among the approximately 690,000 AIDS-related deaths in 2019. In the past two decades, AIDS mortality rates in adolescents observed stagnation or even rise until 2008 but since then declined worldwide similarly with ESA and WCA being the hardest hit regions (Fig. 3b). AIDS-related mortality rate is lower in adolescents than in children, and in contrast, no sex difference is observed. Adolescents’ knowledge, attitude and behavior towards HIV is imperative for ensuring their lifelong protection, and the nations display widely diverse but highly correlated patterns of HIV/AIDS awareness indicators between male and female adolescents (Fig. 3c). Notably, males have higher tendency of condom usage under unsafe sexual practices than females; comprehensive knowledges of HIV are cautiously low (below 50%) in the majority of countries; coverage of HIV testing is limited, and on average, males are less likely to get tested than females.

**Fig. 4:**
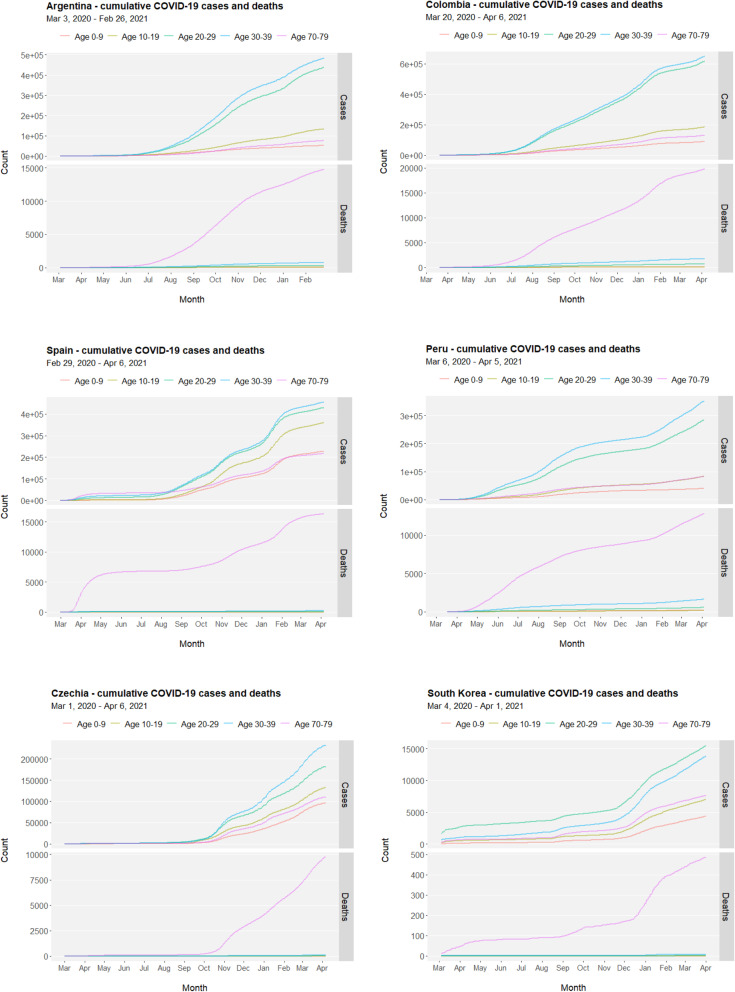
Age-structured COVID-19 cases and deaths. Cumulative cases and deaths in children (aged 0–9), adolescents (aged 10–19), youths (aged 20–29), adults (aged 30–39), and seniors (aged 70–79) in selected countries (Argentina, Colombia, Spain, Peru, Czechia and South Korea) where age-structured information on COVID-19 cases and deaths are available in COVerAGE-DB (https://osf.io/mpwjq/, last accessed on April 7, 2021). Time ranges are 03/03/2020–02/26/2021 for Argentina, 03/20/2020–04/06/2021 for Colombia, 02/29/2020–04/06/2021 for Spain, 03/06/2020–04/05/2021 for Peru, 03/01/2020–04/06/2021 for Czechia, and 03/04/2020–04/01/2021 for South Korea

In a list of countries (Argentina, Colombia, Spain, Peru, Czechia and South Korea) where data of age-structured COVID-19 cumulative cases and deaths are available from COVerAGE-DB, both children and adolescents are relatively less prone to be infected with COVID-19 but their cumulative cases co-fluctuate with other age groups (Fig. 4, upper facets). In comparison, deaths caused by COVID-19 are predominantly concentrated in the senior group (Fig. 4, lower facets). Nevertheless, adolescents worldwide face high COVID-19 risks and disease burdens, particularly in the regions and nations hit hardest by the pandemic and without effective access to vaccine immunization.

## Discussion

Even with incomplete coverage on all nations/territories (the developing countries are better represented than the developed countries), the UNICEF Data warehouse provides a unique platform containing the most accessible and comparable datasets to analyze the cross-sectional levels and longitudinal trends on health indicators. Early-year education and development are instrumental in that children and adolescents falling behind during the early years often never catch up with their peers and thus become more vulnerable to underachievement. Primary education paves the foundation of lifetime learning for the children. Adolescence marks the transition of health and development from childhood to physical and psychological maturity, and much more progress is required in democratizing secondary education worldwide particularly in the least developed regions and nations. Better access and quality of secondary education would lead to transformative changes, equipping the unattended adolescents with knowledge and skills essential for improved employment prospects, reducing adolescent rates in marriage, childbearing and mortality, and preparing more teenagers for the opportunity of higher education that would potentially bring even faster changes to the individuals, families and communities in the time range of just one generation [20].

In this world, inequality is almost everywhere but potentially higher in adolescents who experience disparity due to multiple factors since the start of the life. Gender difference is apparent in health indicators where sex functions as an influential factor (e.g. marriage, childbearing, education, AIDS incidence rate etc.) [21]. The health indicators are interconnected so that gender inequality in one indicator would have chained and amplified consequences on other indicators. For example, early marriage is significantly higher in females (particularly in poor, rural and disadvantaged girls), so child brides are more likely to have pregnancy during adolescence, putting their health at the highest risks with the least antenatal and maternal care. Raising children when they are themselves not mature adults, adolescent mothers may suffer from physical or mental diseases, terminate education prematurely and permanently, allocate limited resources unfairly to the children (e.g. with preference to boys), and ultimately reinforce the unending cycles of gender inequality. Policies promoting, legalizing and executing girls’ equal access to education, healthcare and socioeconomic opportunities are urgently required for achieving gender equality and empowering all women and girls (SDG Target 5). For other indicators of which gender disparity is low, the differences conferred by socioeconomic conditions and living locations could be significant, making adolescents from the poorest rural households in least developed regions and nations the most vulnerable and underprivileged group [22, 23]. Ironically, this group, to whom assistance and intervention is theoretically most needed, is in reality often either passively shunned in the surveys given the difficulty of coverage [24], or unintentionally ignored during the averaging summary of the larger population [25]. Even within nations, subnational disparities are also widely present but extremely difficult to survey and evaluate in a systematic scale, further compounding the underlying inequalities within the health of adolescents. Again, due to the limited data coverage, it is noteworthy that the discoveries on inequality and disparity in this study are largely relevant to those (mostly developing) countries and regions for which data are present and analyzed. It is equally important to aim for better data coverage and more analysis of those countries not yet covered by UNICEF Data for the status of inequality.

The fight against HIV requires separate approaches and strategies in children compared to adolescents. New HIV infections in children are predominantly caused by mother-to-child transmission, so interventions including HIV-status diagnosis before pregnancy, lifelong antiretroviral treatment for HIV-positive women and the preventive administration of antiretroviral therapy in their exposed newborns together would lead to a possible control on the HIV epidemic in children. In contrast, new HIV infections in adolescents arise due to risky behaviors (e.g. unprotected heterosexual or homosexual activities, shared drug injections etc.) that can be intervened (e.g. sex education, condom usage, drug control etc.) [26]. Scaling up access to the prevention and treatment of HIV in the young generation is consistently overdue as the world miserably failed its ambitious 90–90-90 goals by 2020 (90% of the HIV-infected knowing their status, 90% of the knowing HIV-infected on treatment, and 90% of the on-treatment HIV-infected virally suppressed and sustained) [27].

First appearing at year-end 2019, COVID-19 scorched the world throughout 2020 and the ongoing pandemic continued into 2021 waiting for the newly developed vaccines to finally press a pause or even an end to the public health crisis. The long-term risks of COVID-19 on the health of adolescents are not yet fully understood [28] as the relevant data remain to be aggregated and released, but it could be robustly inferred that this unexpected and destructive pandemic has a long-lasting and consequential impact on almost every aspect of human society and civilization. Directly, COVID-19 infections result in unacceptably high counts of cases and deaths, possibly raising overall mortality in those hardest hit regions and nations. With lessons from past West Africa Ebola [29], MERS-CoV [30] and SARS [31] epidemics, it is reasonably possible that the indirect impacts of COVID-19 could outpace the direct costs of the pandemic. National/regional lockdowns, travel restrictions and social distancing regulations are expected to undercut education coverage, exacerbate mental health issues, and cause family income loss, depriving adolescents of their familiar environment of personal development. In the least developed regions and nations, decades of progress on the reduction of early marriage and childbearing are under threat. Diversion of limited healthcare resources to COVID-19 testing and treatment would disrupt access and quality of pediatric care, routine immunization services, HIV testing and supply of antiretroviral medications, community health management, and other essential health services [32]. Most importantly, the pandemic is disproportionally hitting the most marginalized and underprivileged subgroups, substantially exacerbating the established inequality to a potentially historic level [33].

Given the scope and severity of the COVID-19 pandemic, it is theoretically guaranteed that the health indicators on adolescents analyzed here on global, regional and national levels until 2019 should deteriorate when the latest UNICEF data on 2020 and beyond are released in the near future. It is time to fight toward the restoration of adolescent health status to pre-pandemic levels before aiming to reach the far-fetched SDG targets.

## Supplementary Information

**Additional file 1.**

## Data Availability

Data of health indicators are publicly accessible from the UNICEF Data warehouse (data.unicef.org/dv_index/). Data of age-structured COVID-19 cases and deaths are publicly accessible from the COVerAGE-DB (https://osf.io/mpwjq/).
