# Correction to: Health indicators on adolescents reveal disparity and inequality on regional and national levels

**DOI:** 10.1186/s12889-021-12155-z

**Published:** 2021-11-15

**Authors:** Mengqiao Wang

**Affiliations:** Department of Epidemiology and Biostatistics, West China School of Public Health and West China Fourth Hospital, Renmin South Road 16, Chengdu, Sichuan Province 610041 People’s Republic of China

**Correction to: BMC Public Health 21, 1006 (2021)**

**https://doi.org/10.1186/s12889-021-10989-1**

It was highlighted that in the original article [1] the Funding section was incomplete. This Correction article shows the correct Funding section. The original article has been updated.

**Funding**

Research by M.W. was supported by the Youth Scholar Grant from the National Natural Science Foundation of China (81800071) and the Basic Research Grant from the Department of Science and Technology of Sichuan Province (2019YJ0035). The funding agency has no direct role in the study design, the data analysis, and the writing/editing of manuscript.
