# Supplementary material for: Health indicators on adolescents reveal disparity and inequality on regional and national levels
Source: BMC Public Health. 2021 May 28;21:1006. doi: 10.1186/s12889-021-10989-1 (PMC8161722; doi:10.1186/s12889-021-10989-1)
Supplement: Supplementary file 1 — Additional file 1. [file 12889_2021_10989_MOESM1_ESM.docx]

**Health indicators on adolescents reveal disparity and inequality on regional and national levels**

Mengqiao Wang*

Department of Epidemiology and Biostatistics, West China School of Public Health and West China Fourth Hospital, Sichuan University, P.R. China

Address: Renmin South Road 16, Chengdu, Sichuan Province, 610041, P.R. China.

Phone: 86-13880649045

* Corresponding author ([mengqiaowang@gmail.com](mailto:mengqiaowang@gmail.com))

**Supplemental materials**

- 11 supplementary figures

**Supplementary Figures**

**Figure S1**

A.


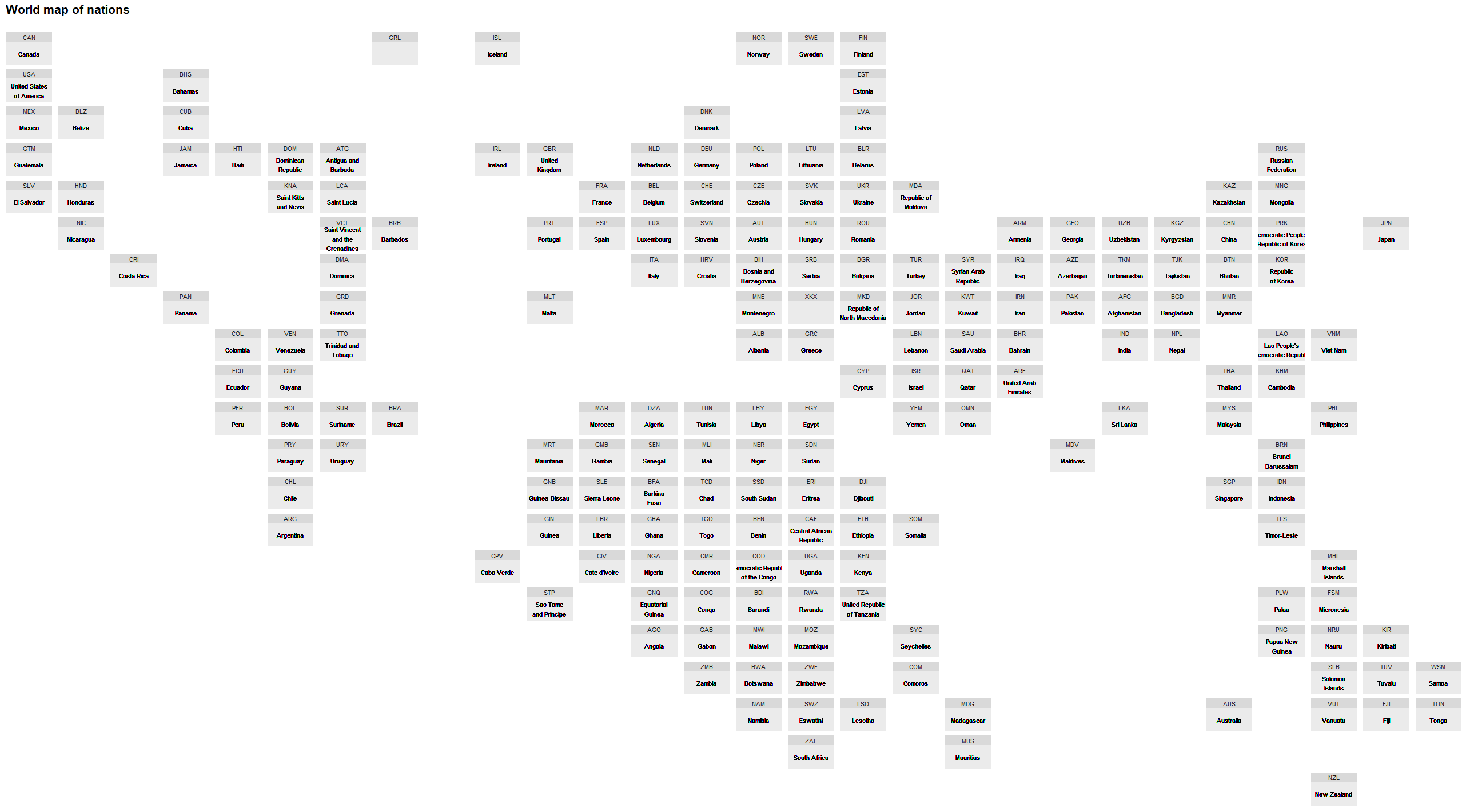


B.


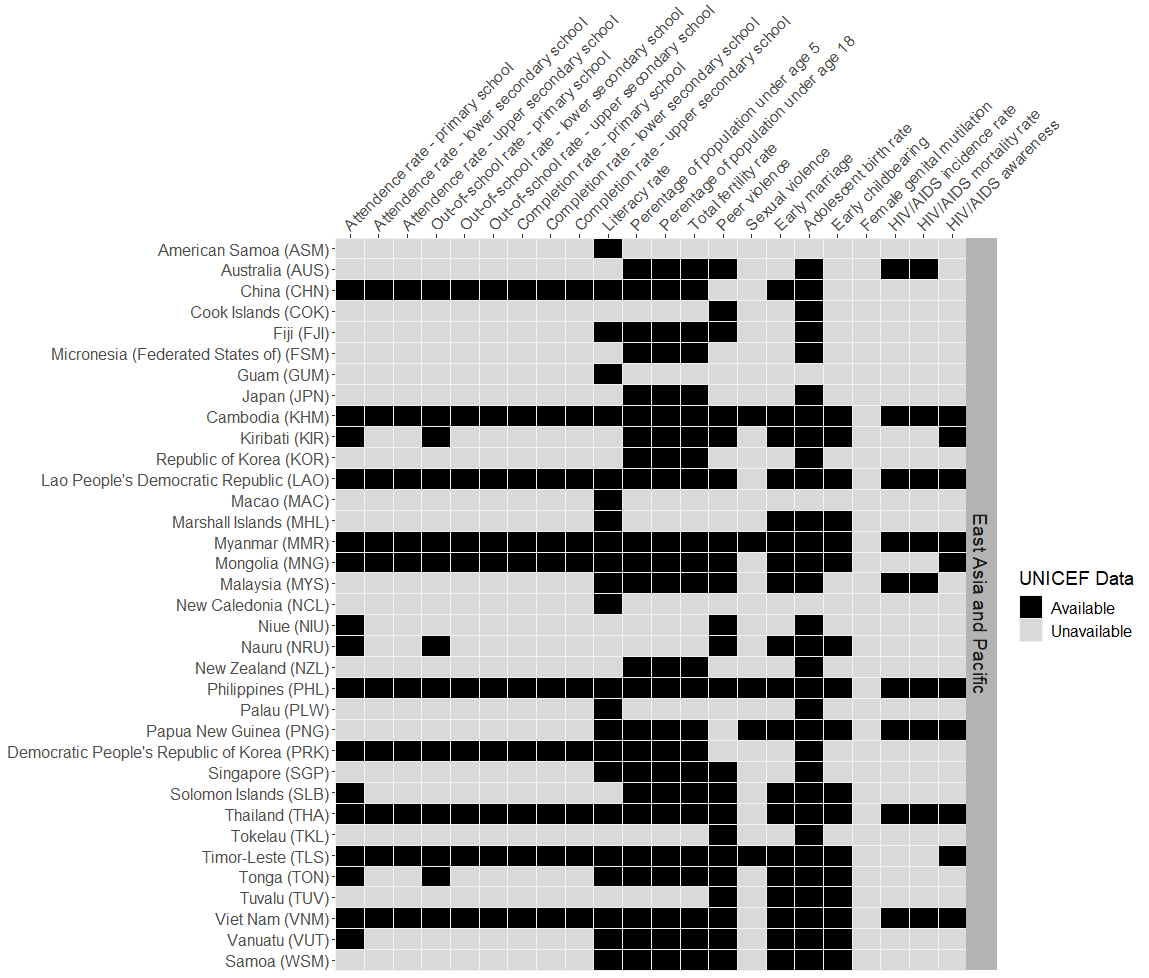


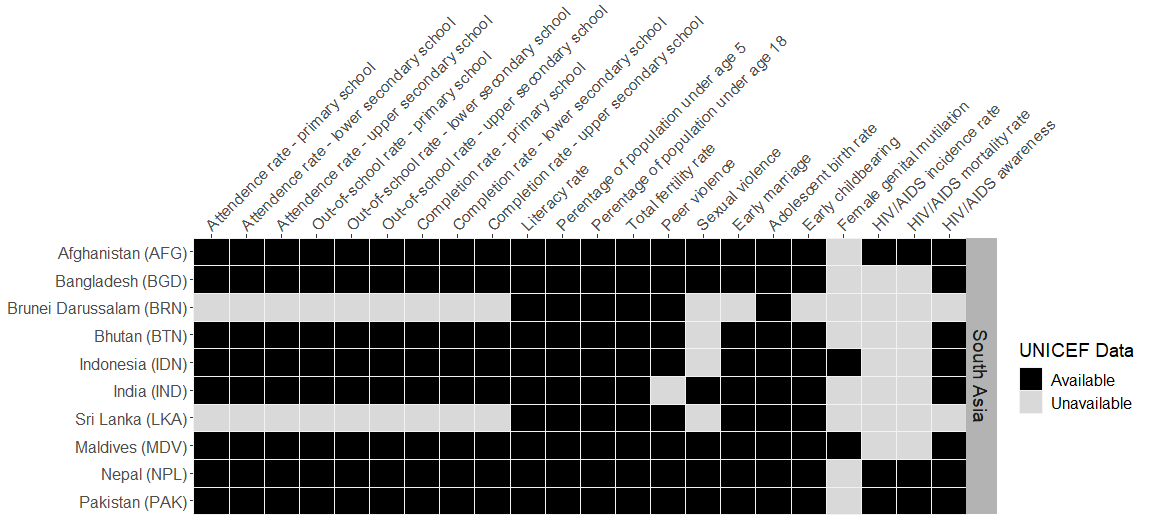


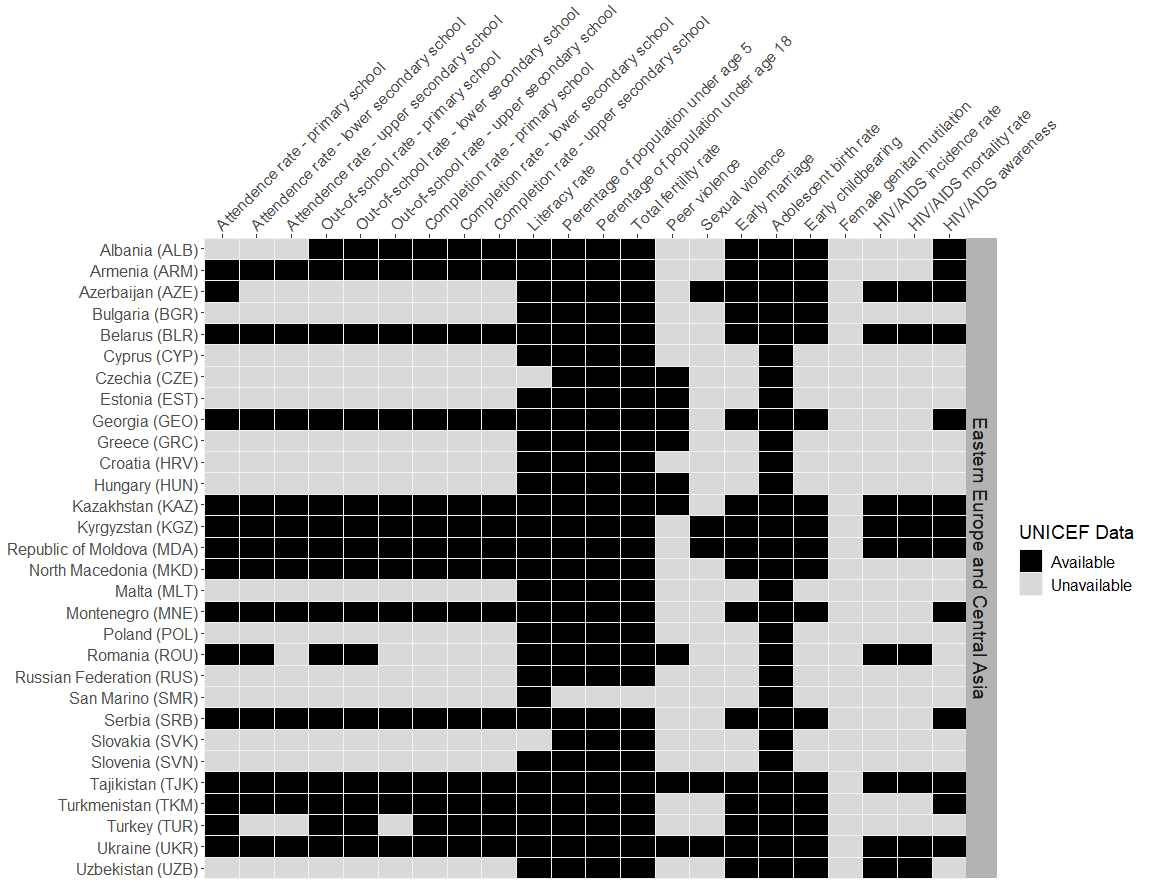


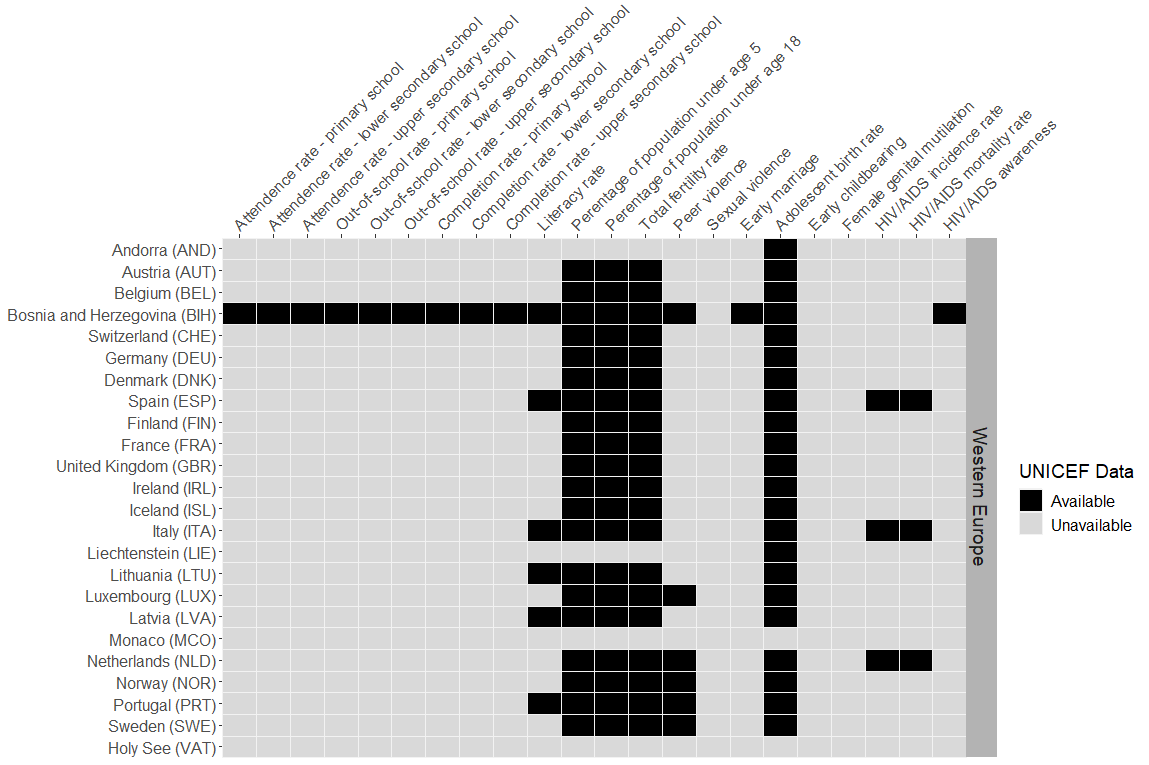


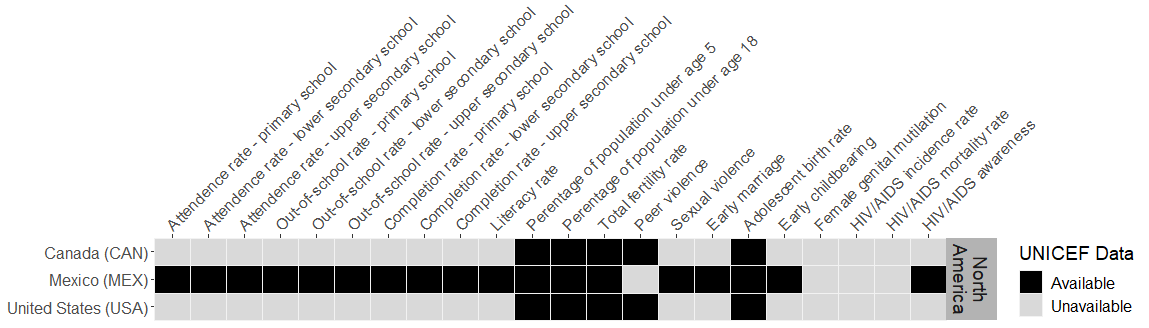


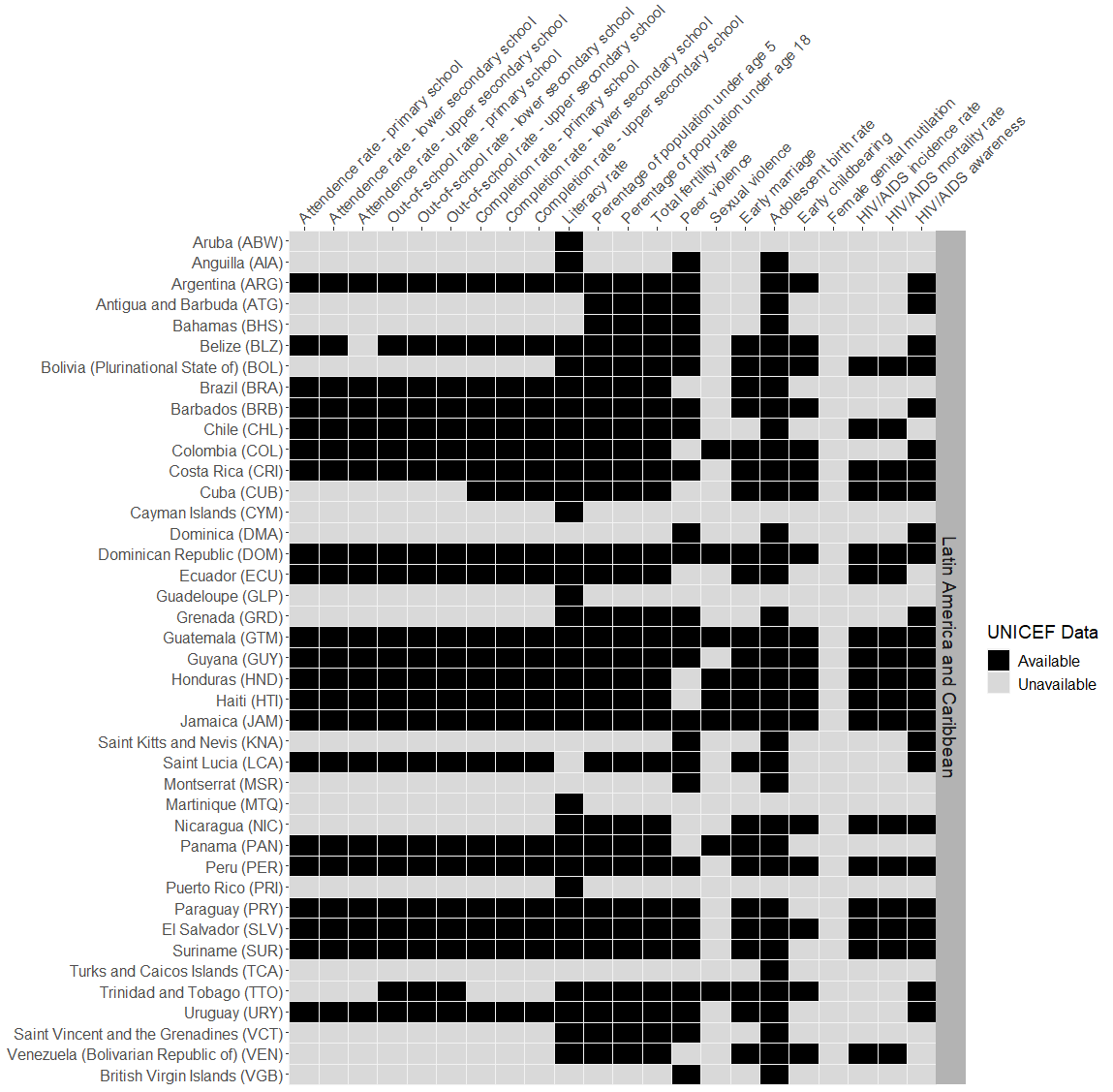


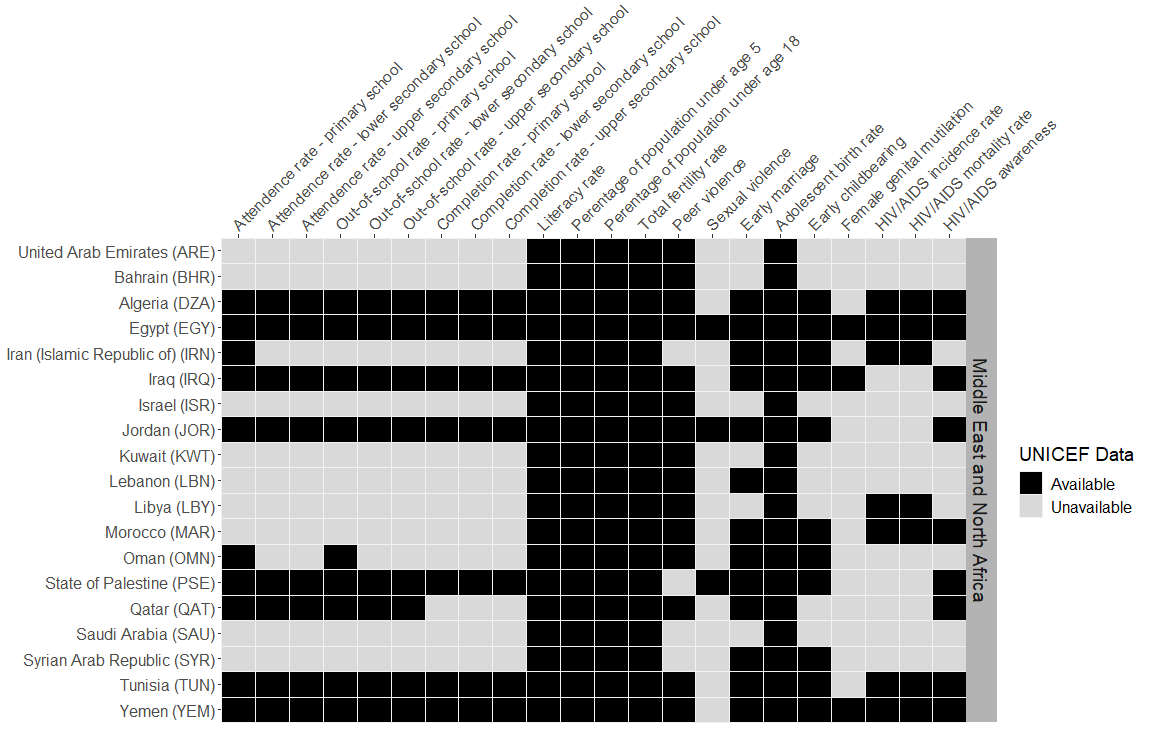


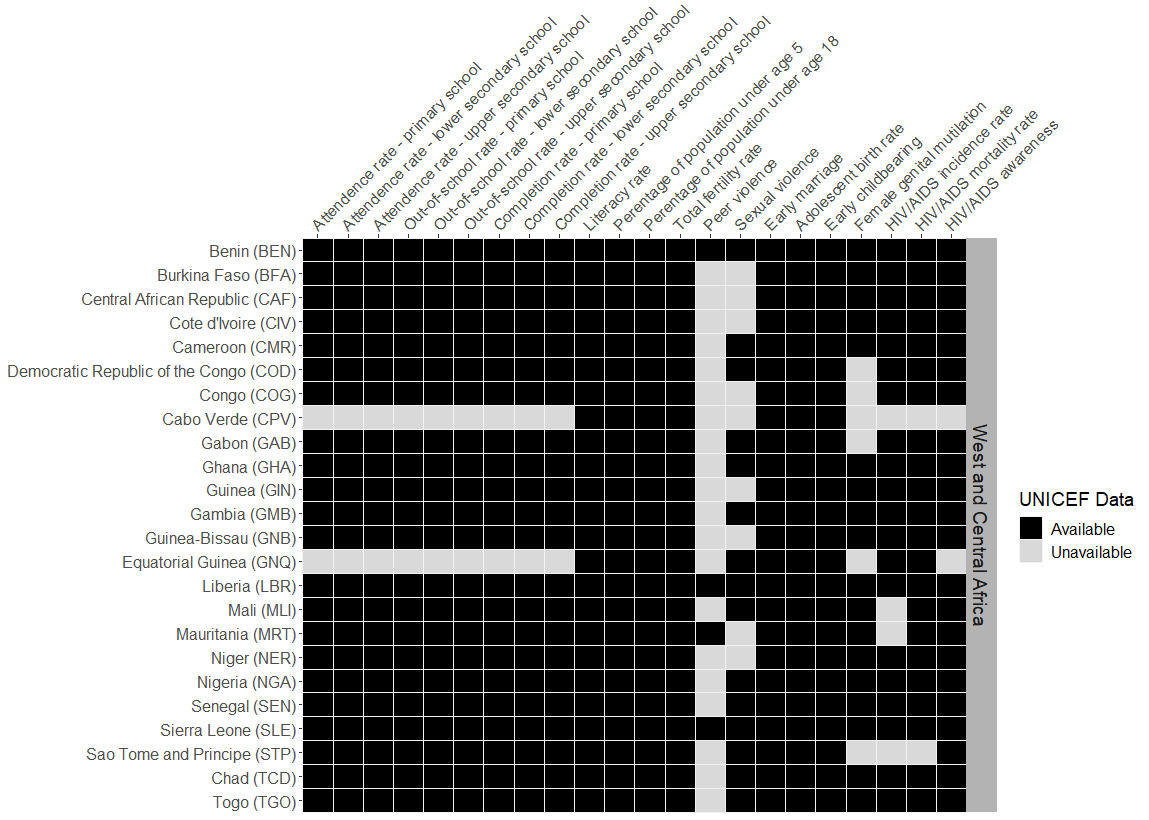


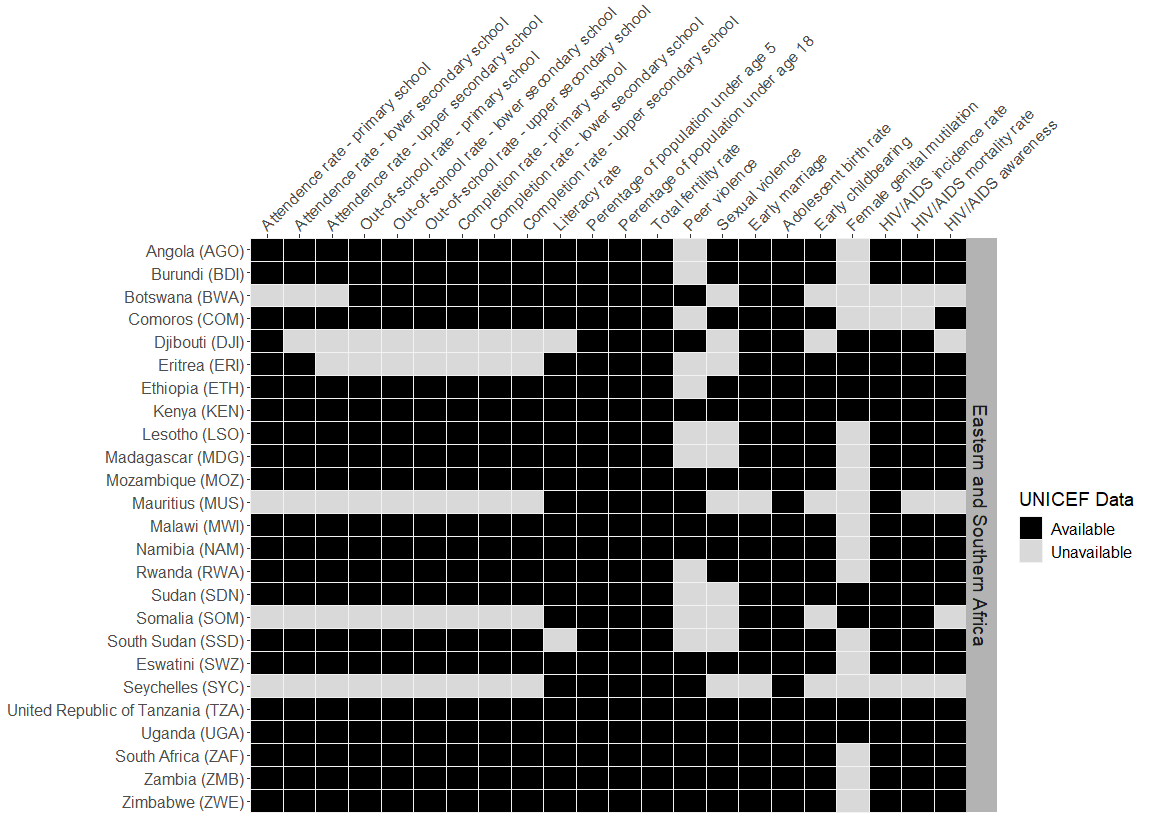


**Figure S2**


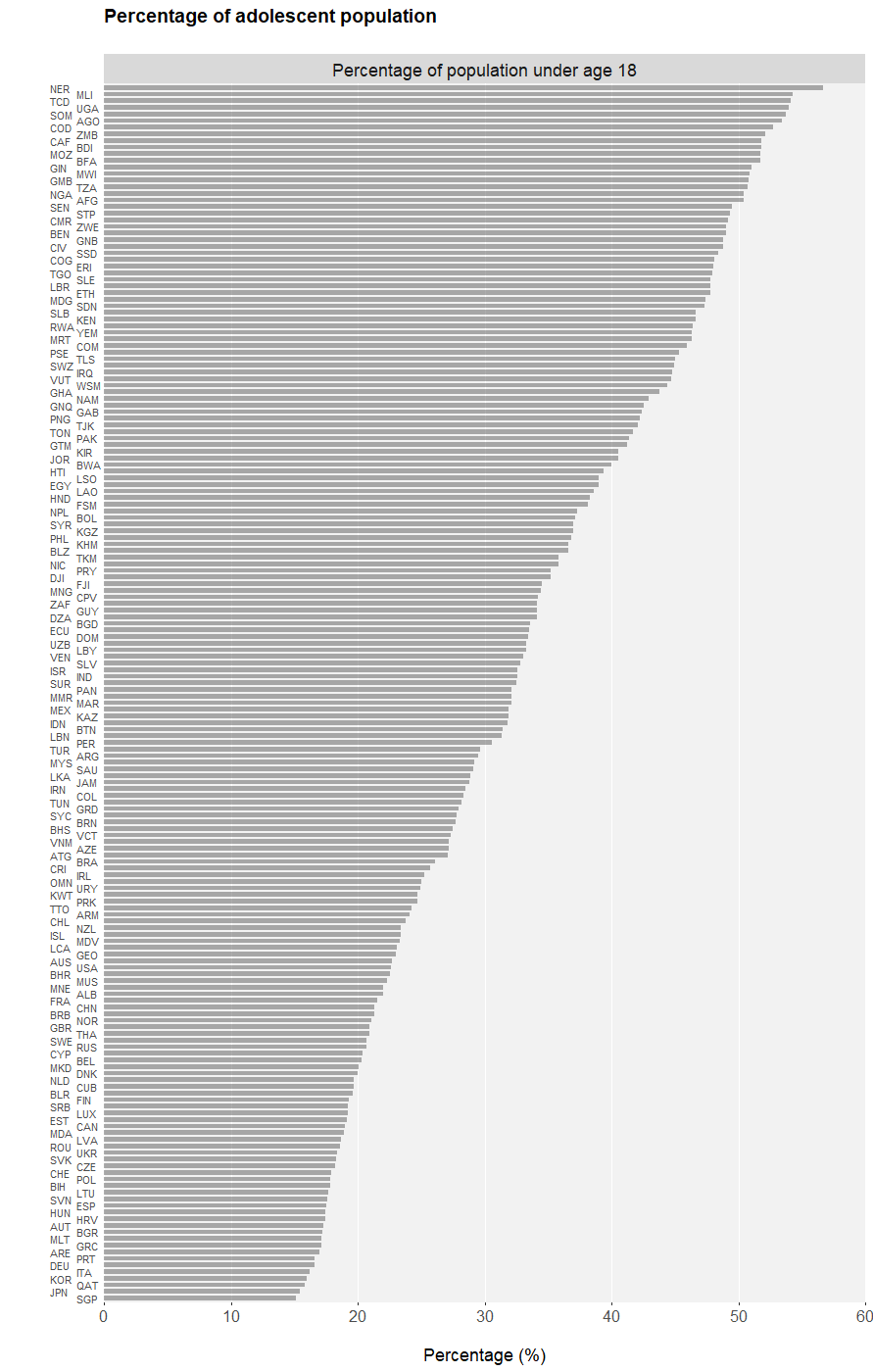


**Figure S3**


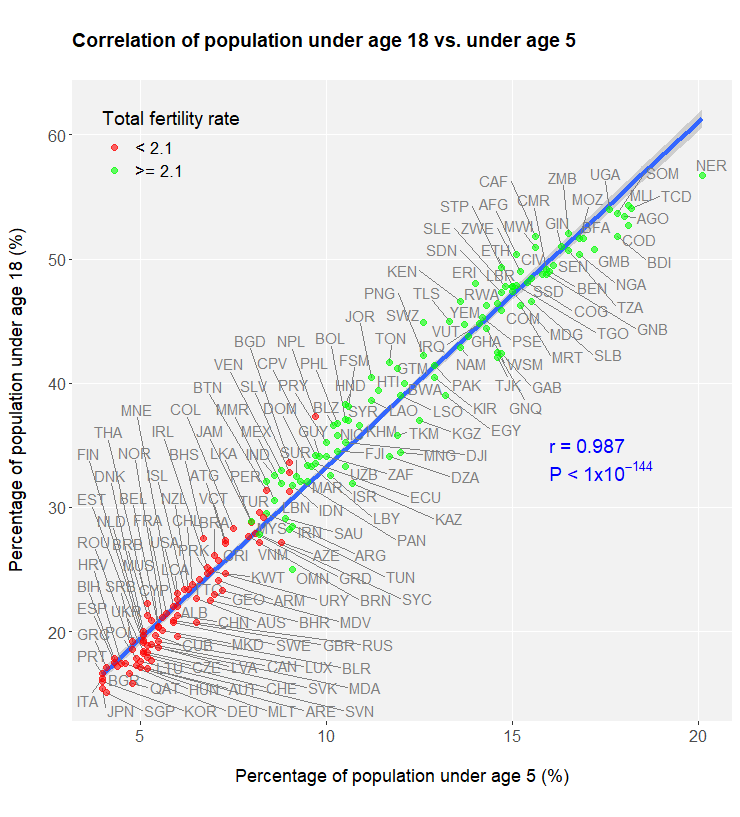


**Figure S4**


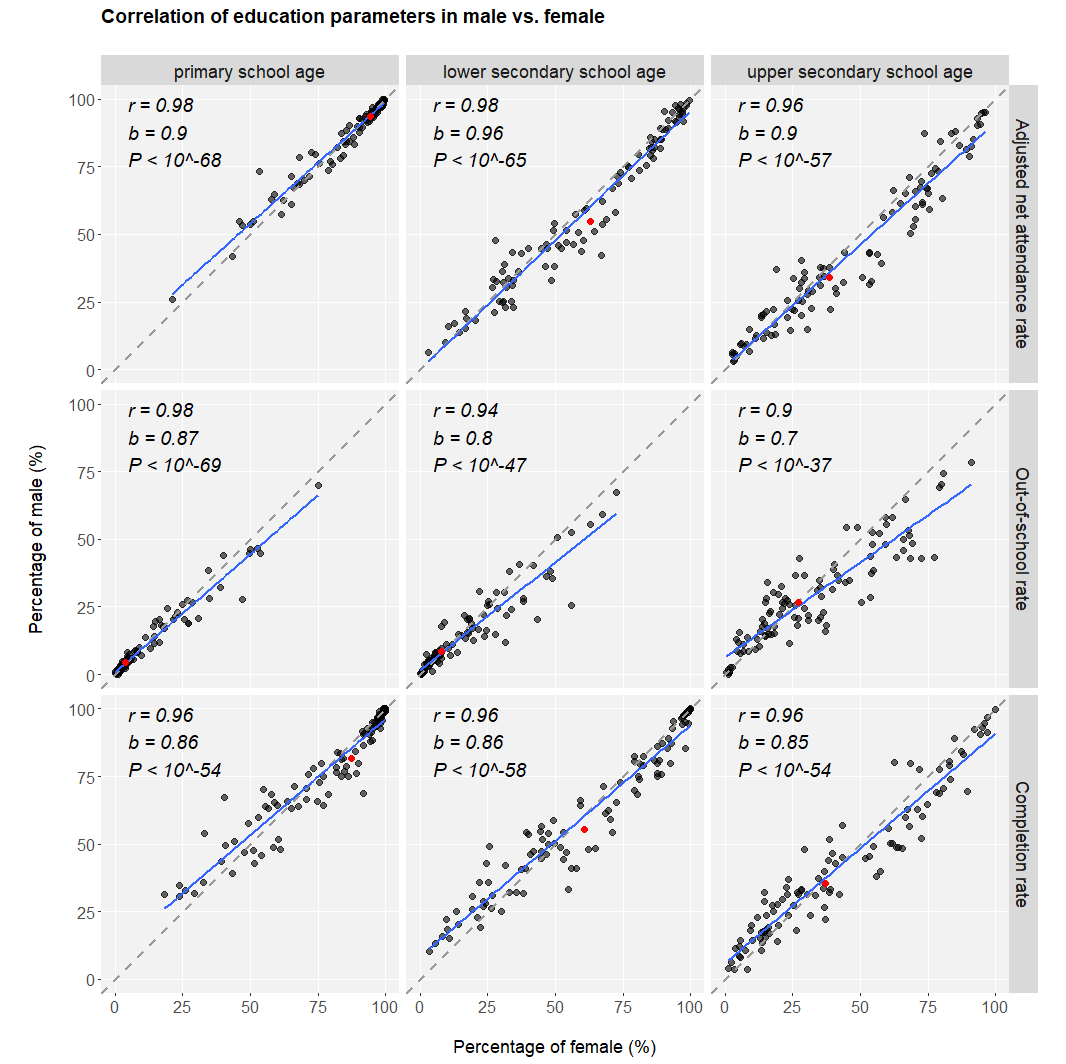


**Figure S5**


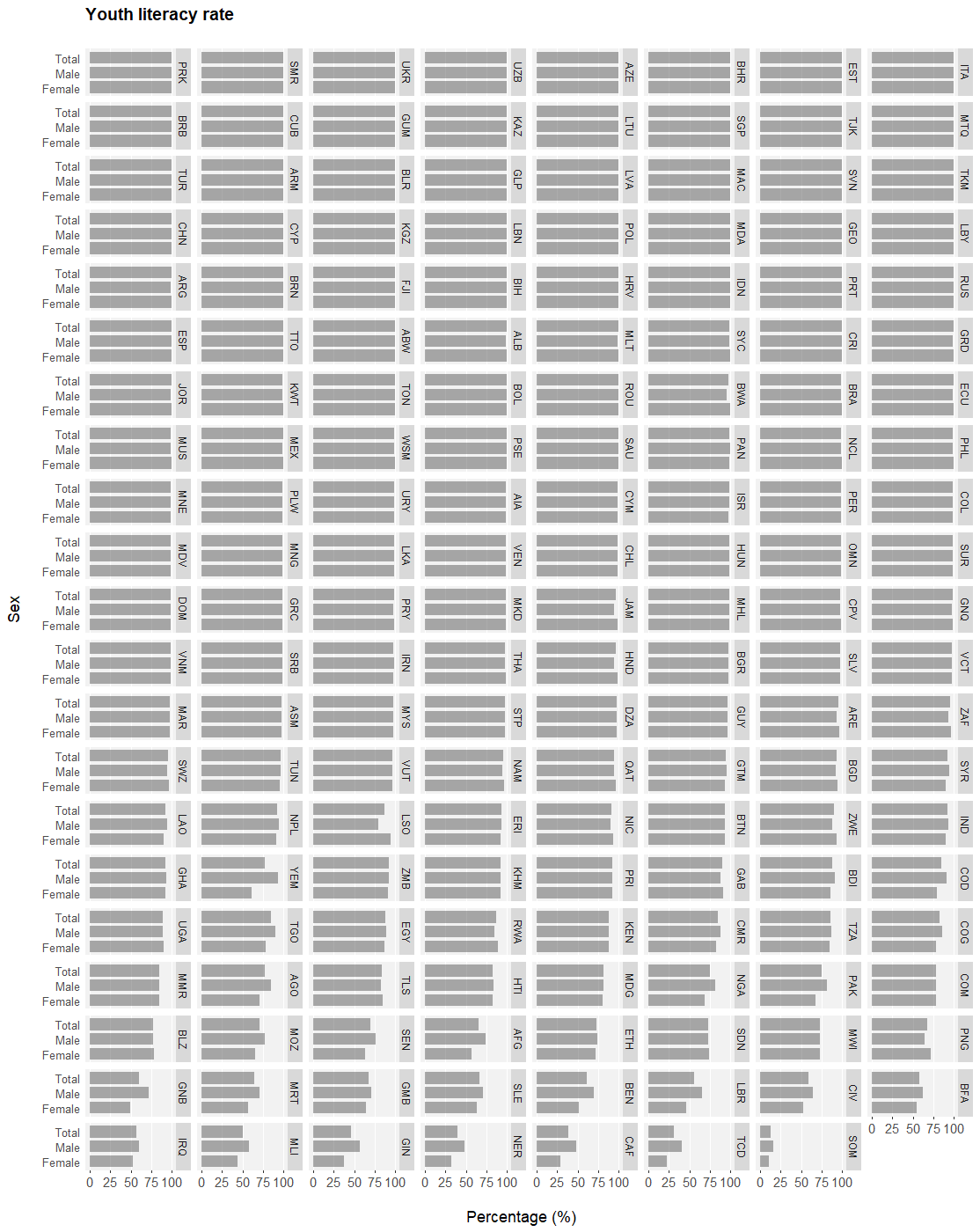


**Figure S6**


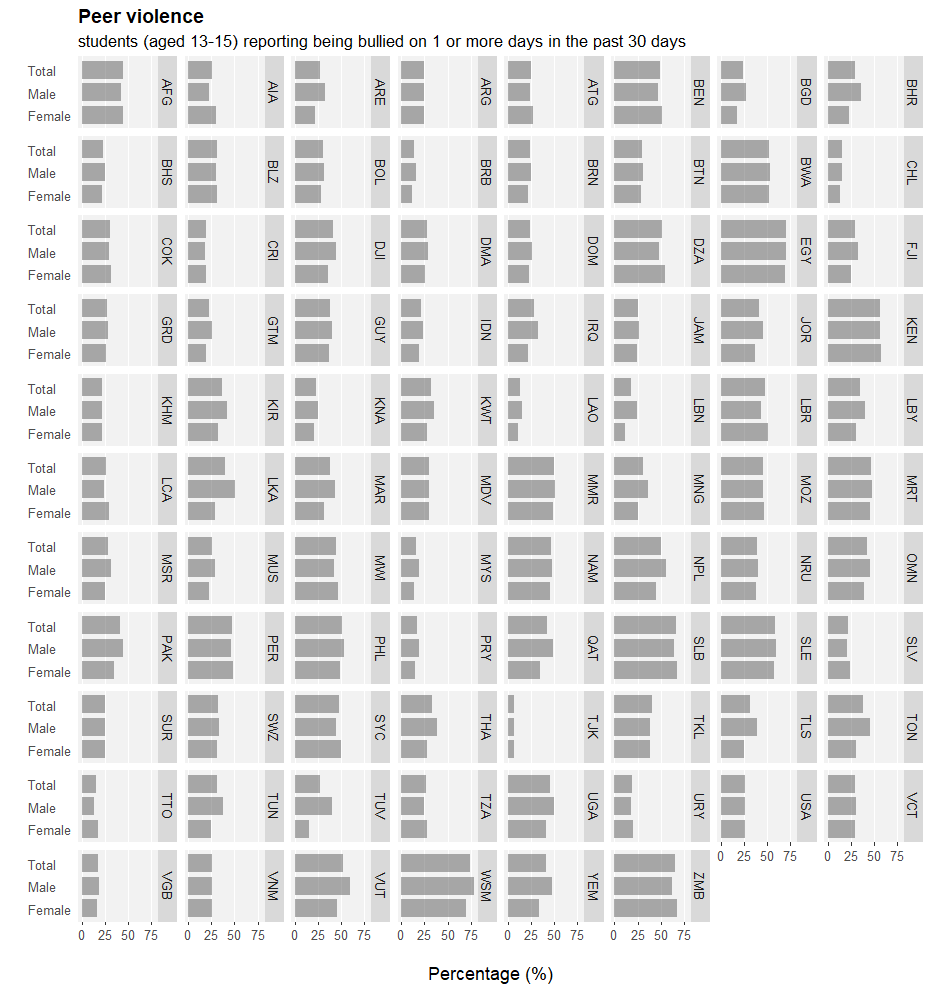


**Figure S7**


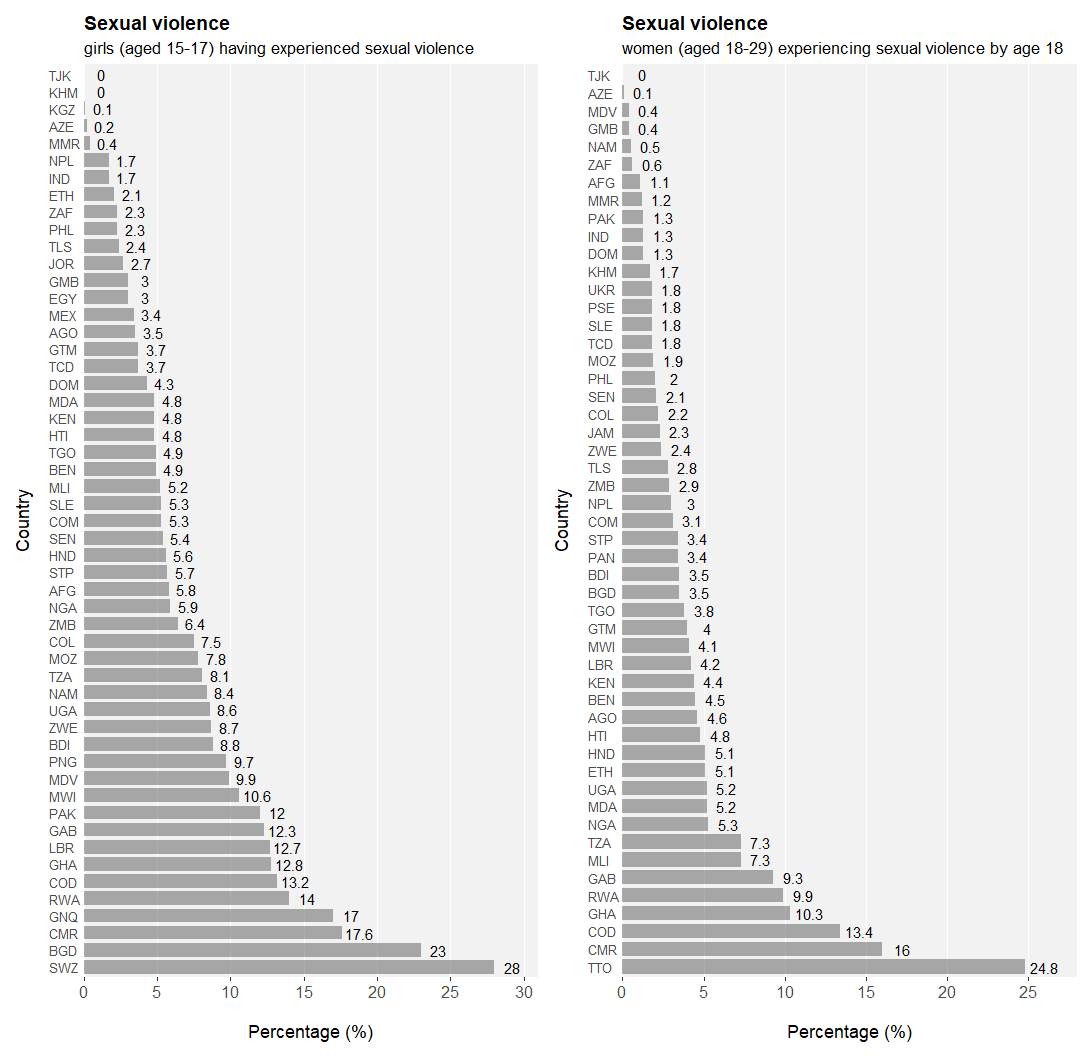


**Figure S8**


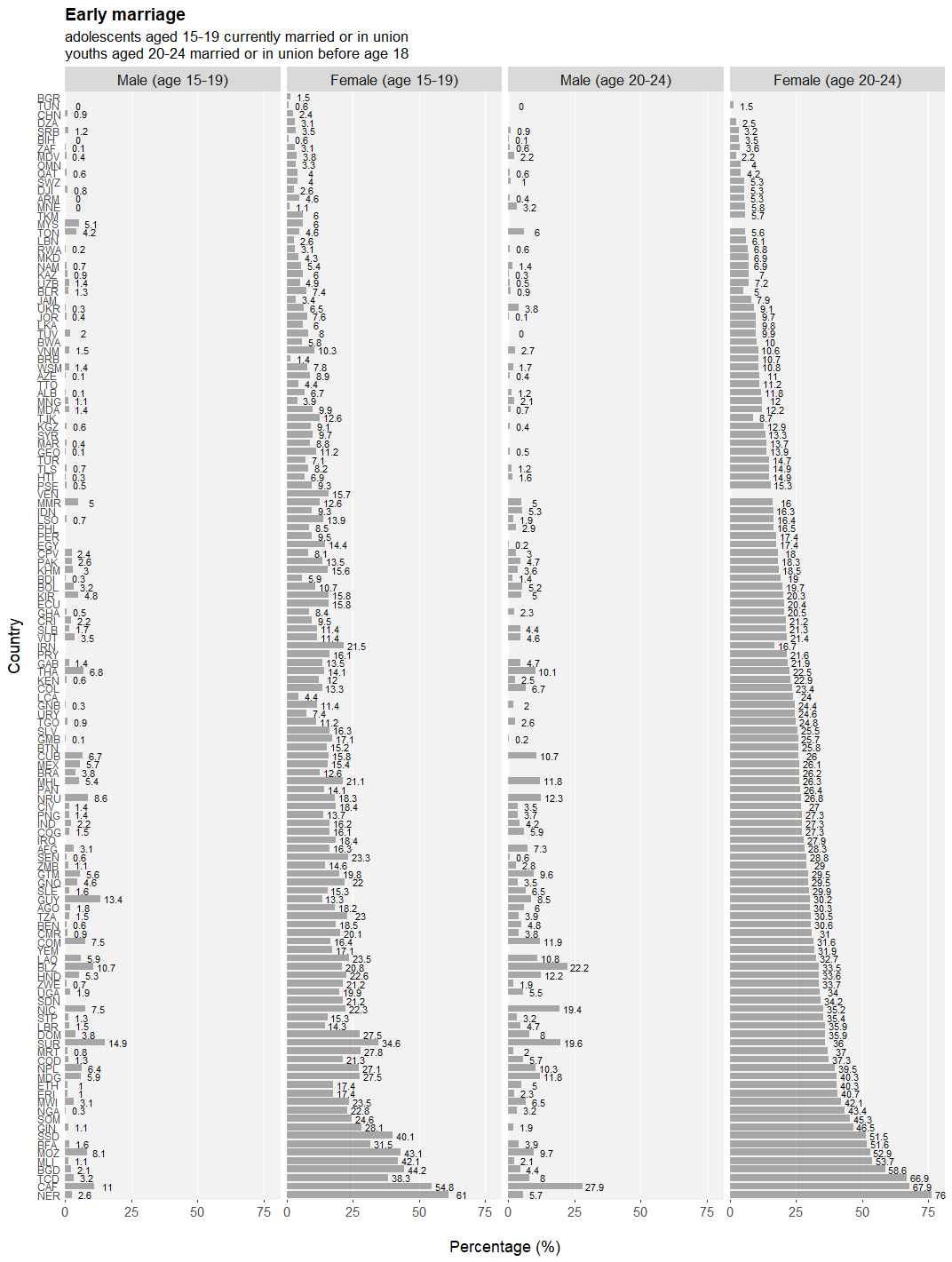


**Figure S9**

A.


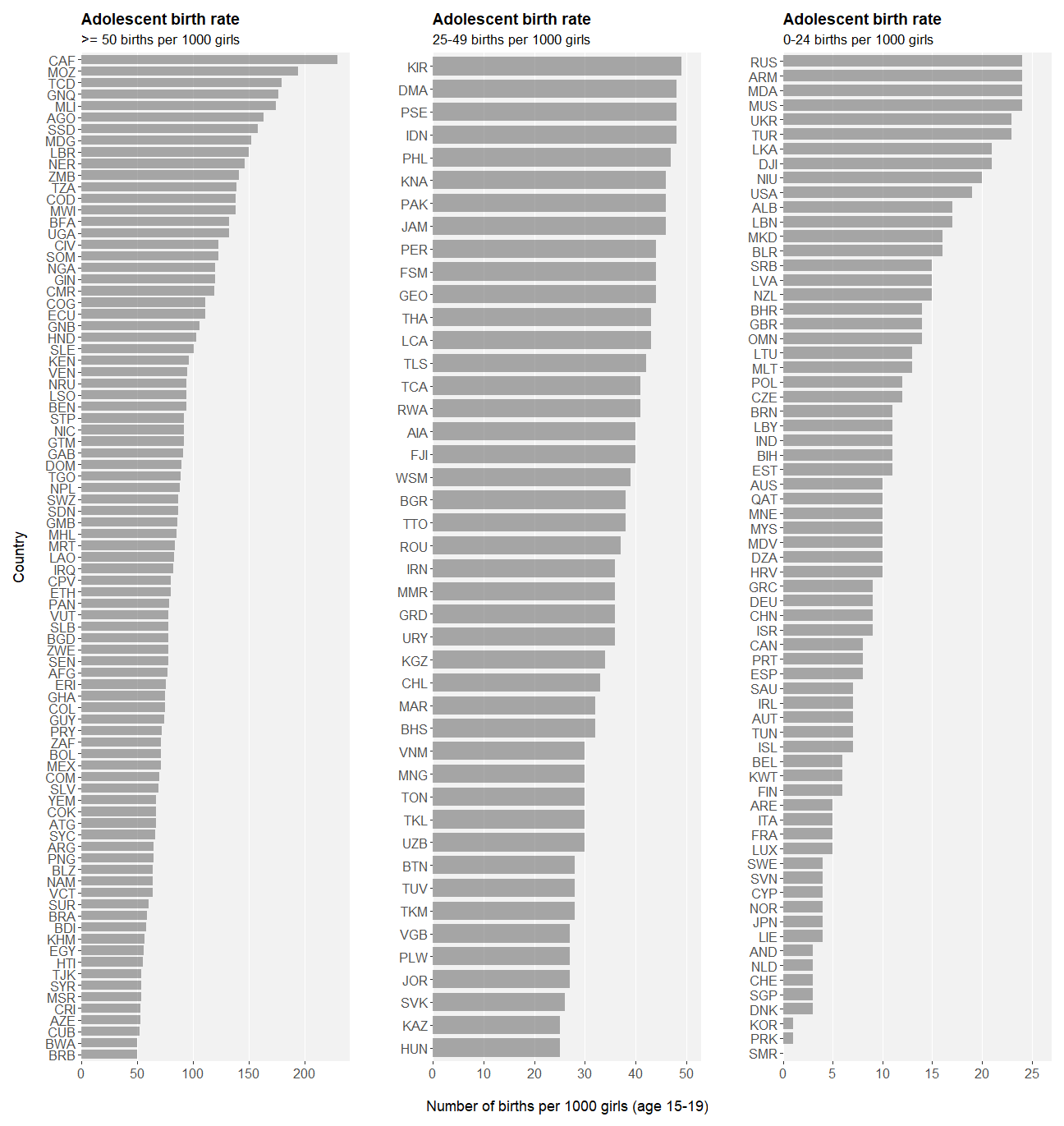


B.


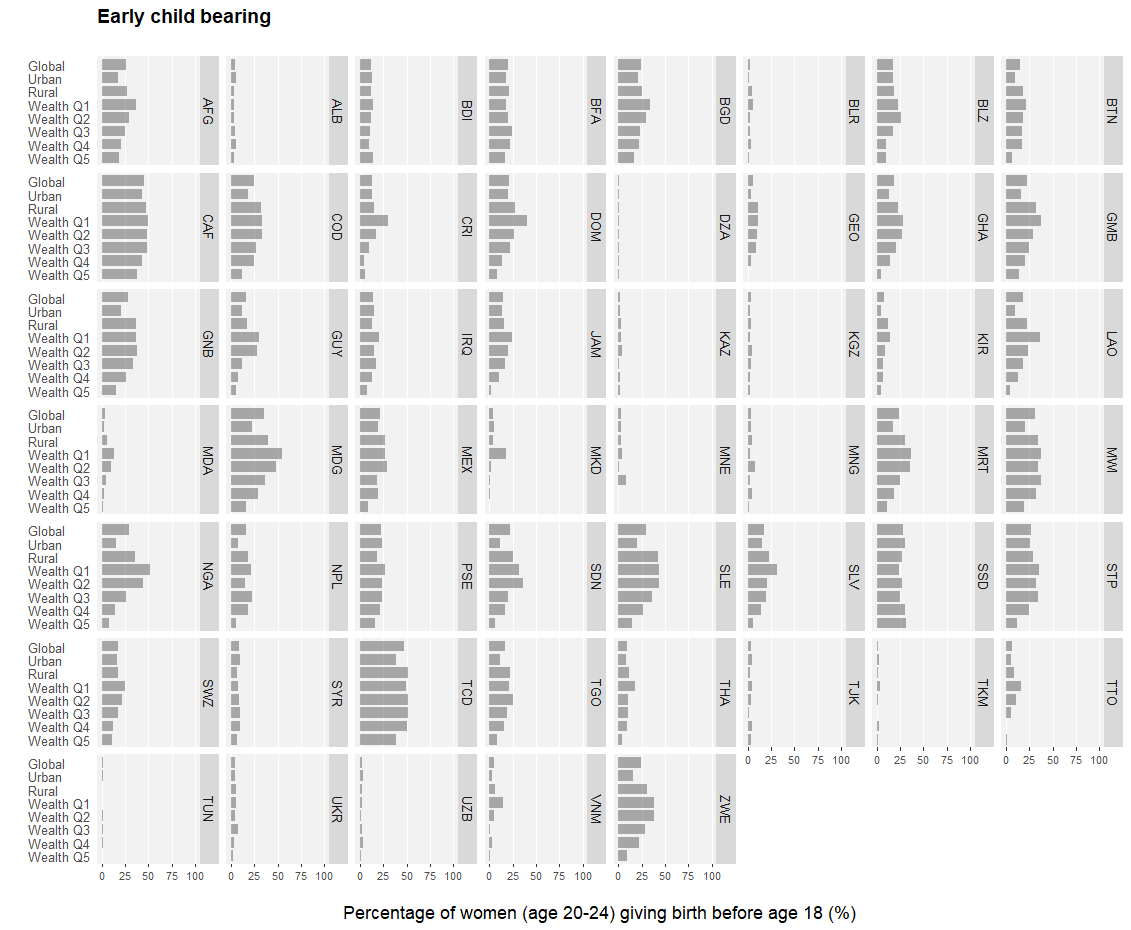


**Figure S10**


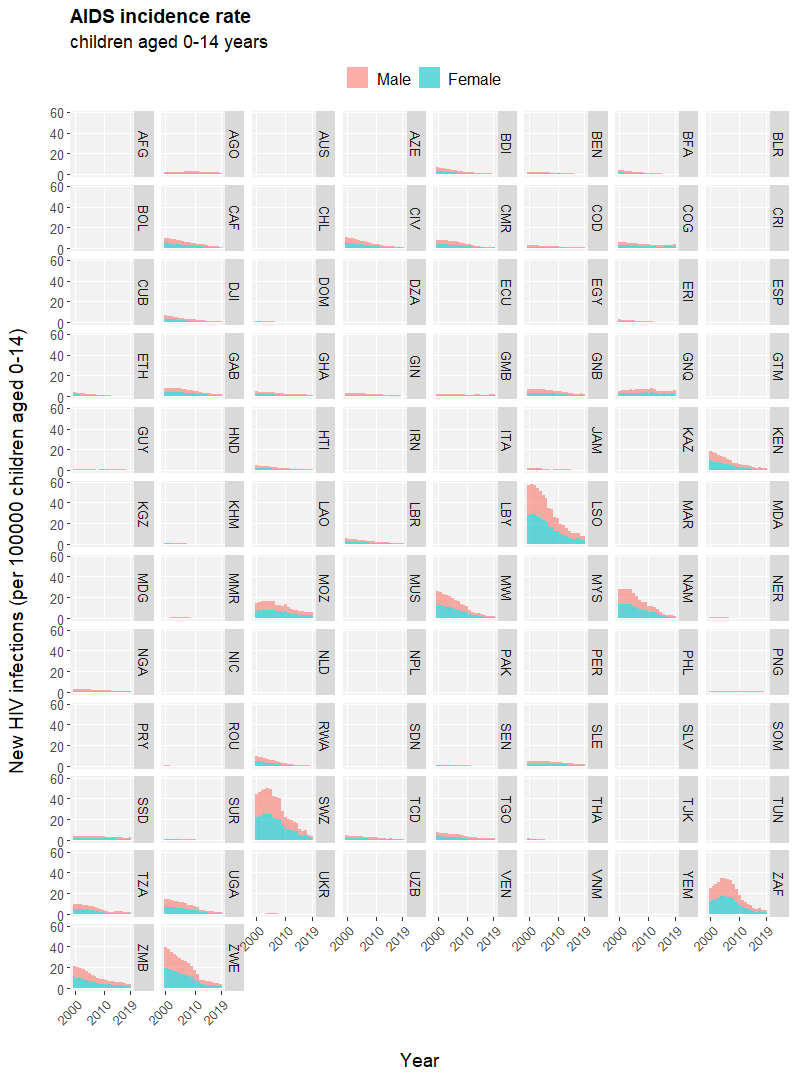


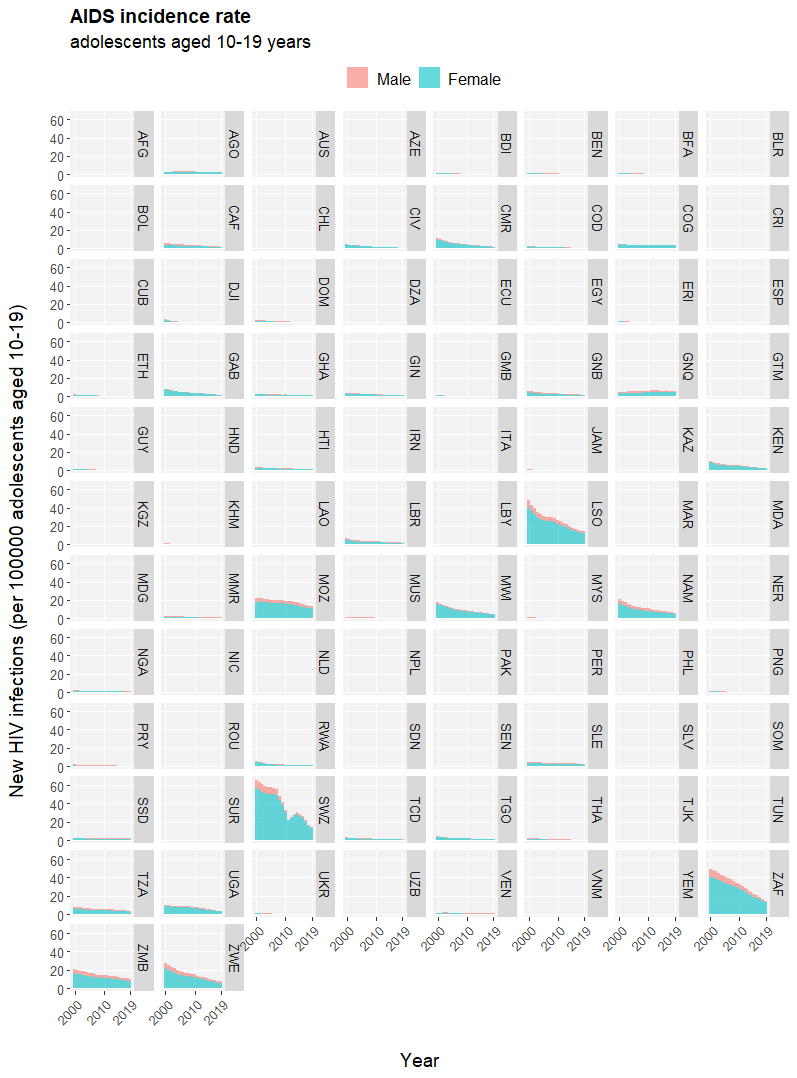


**Figure S11**


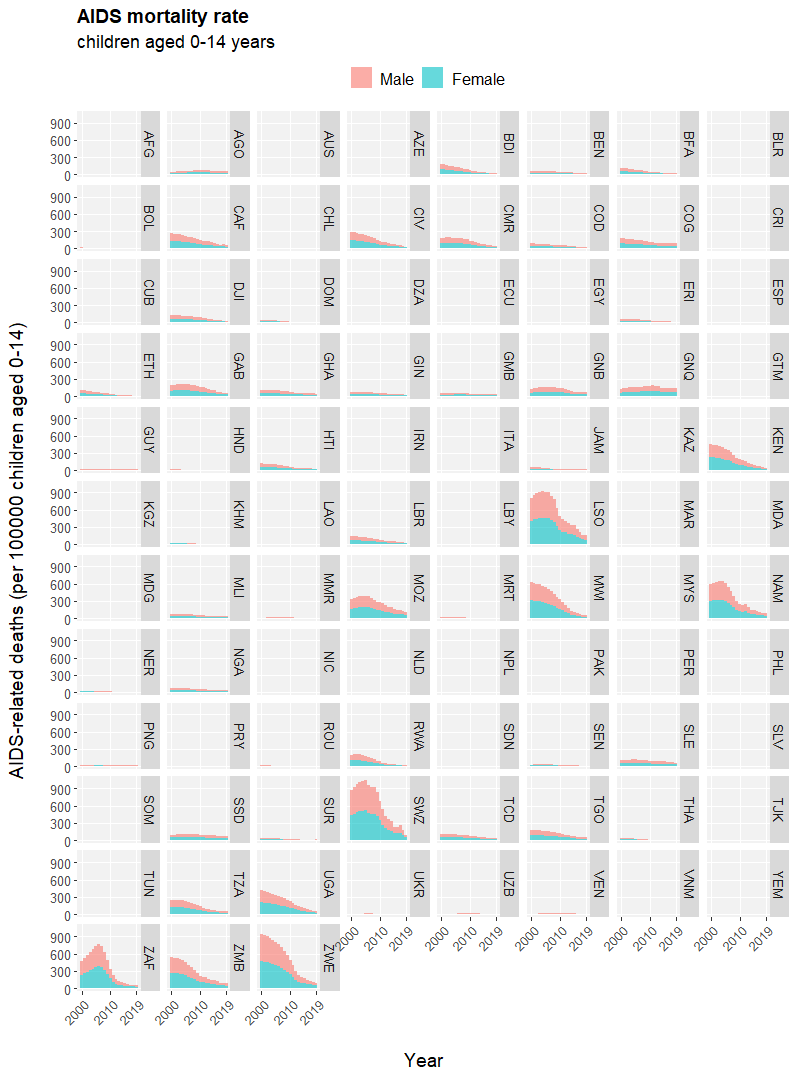


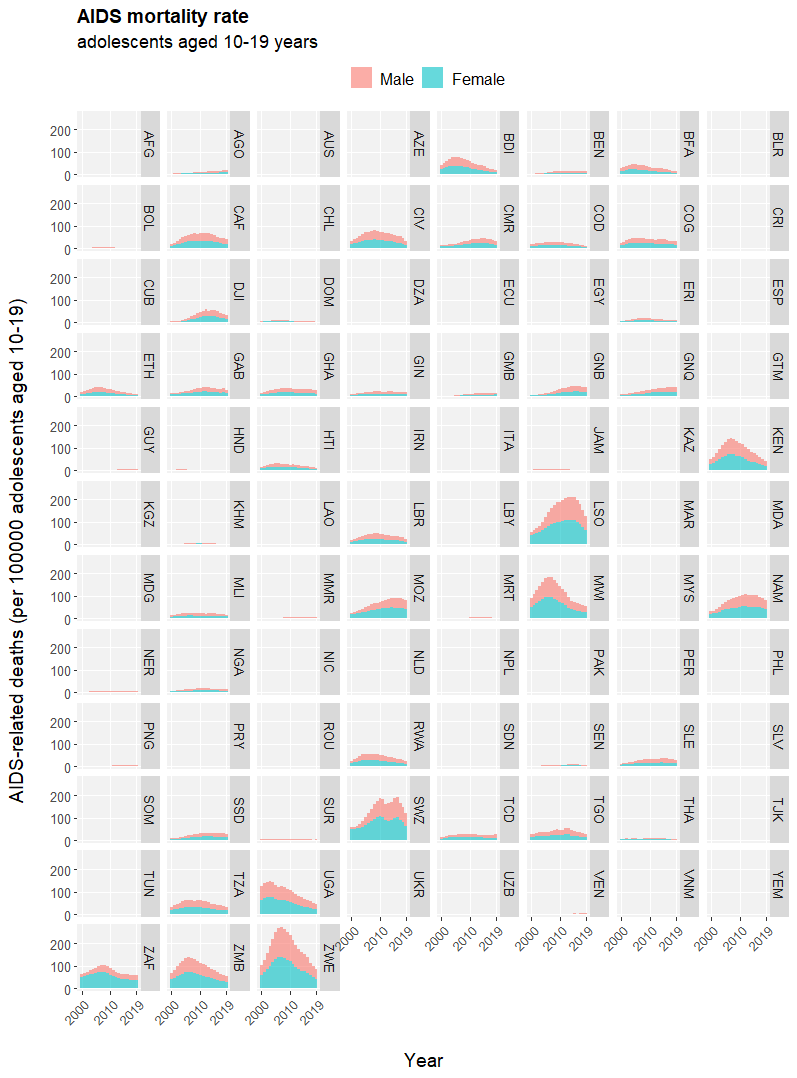


**Supplementary Figure Legends**

Figure S1. A. World map of countries/territories with names and ISO3 codes displaying their relative geographic locations. B. Description of data availability of UNCIEF health indicators included in this study for all countries/territories by the respective regions.

Figure S2. Proportion of under-18 (adolescents) population out of total population among countries in 2018. *Data source: United Nations Population Division (UNPD), 2019.*

Figure S3. Scatter plot of under-5 (children) and under-18 (adolescents) population proportions among countries in 2018. The trend line fitted by linear regression is displayed as a blue solid line with the associated Pearson’s correlation coefficient (*r*) and null-hypothesis test P value (*P*) reported. Points are shaded with regard to the status of total fertility rate (TFR). 2.1 is the commonly accepted threshold for the inter-generational replacement TFR. *Data source: United Nations Population Division (UNPD), 2019.*

Figure S4. Correlation of education status (adjusted net attendance rate, out-of-school rate, and completion rate) for school age (primary, lower secondary and upper secondary) in male *vs.* female among countries. *Data source: UNICEF global databases, 2019.*

Figure S5. Youth literacy rate (percentage of literate persons aged 15-24) conditioned on sex (male and female) among countries. *Data source: UNESCO Institute for Statistics global databases, 2019, based on survey data for the most recent year available during the period 2012-2018.*

Figure S6. Percentage of adolescents (aged 13-15) having underwent peer violence (bullying) conditioned on sex (male and female) among nations. *Data source: HBSC, GSHS, and other relevant surveys, the most recent version.*

Figure S7. Percentage of girls (aged 15-17) having experienced sexual violence and young women (aged 18-29) having experienced sexual violence by the age of 18 among nations. *Data source: DHS and other relevant surveys, the most recent version.*

Figure S8. Percentage of early marriage in adolescents (aged 15-19 and 20-24) by sex (male and female) among nations. *Data source: UNICEF MICS, DHS, and other relevant surveys, the most recent version.*

Figure S9. Status of early child bearing. A. Adolescent birth rates in female adolescents (aged 15-19) among nations. *Data source: UN Population Division 2018.* B. Percentage of women (aged 20-24) giving birth before the age of 18 conditioned on living environment (urban and rural) and economic condition (wealth status in 5 quintiles of 20%) among nations. *Data source: UNICEF MICS, DHS, and other relevant surveys, the most recent version.*

Figure S10. AIDS incidence rates in children (aged 0-14) and adolescents (aged 10-19) by sex (male and female) among nations in the past two decades (2000-2019). *Data source: UNAIDS 2020 estimates.*

Figure S11. AIDS mortality rates in children (aged 0-14) and adolescents (aged 10-19) by sex (male and female) among nations in the past two decades (2000-2019). *Data source: UNAIDS 2020 estimates.*
